# Phylogeny and symbiotic effectiveness of indigenous rhizobial microsymbionts of common bean (*Phaseolus vulgaris* L.) in Malkerns, Eswatini

**DOI:** 10.1038/s41598-023-43634-5

**Published:** 2023-10-09

**Authors:** Rotondwa P. Gunununu, Mustapha Mohammed, Sanjay K. Jaiswal, Felix D. Dakora

**Affiliations:** 1 Department of Crop Sciences, Tshwane University of Technology, Private Bag X680, Pretoria, 0001 South Africa; 2 Department of Chemistry, Tshwane University of Technology, Private Bag X680, Pretoria, 0001 South Africa; 3 Department of Crop Science, University for Development Studies, P.O. Box TL1882, Tamale, Ghana

**Keywords:** Microbiology, Bacteria, Symbiosis

## Abstract

In most legumes, the rhizobial symbionts exhibit diversity across different environments. Although common bean (*Phaseolus vulgaris* L.) is one of the important legumes in southern Africa, there is no available information on the genetic diversity and N_2_-fixing effectiveness of its symbionts in Malkerns, Eswatini. In this study, we assessed the phylogenetic positions of rhizobial microsymbionts of common bean from Malkerns in Eswatini. The isolates obtained showed differences in morpho-physiology and N_2_-fixing efficiency. A dendrogram constructed from the ERIC-PCR banding patterns, grouped a total of 88 tested isolates into 80 ERIC-PCR types if considered at a 70% similarity cut-off point. Multilocus sequence analysis using 16S rRNA, *rpoB, dnaK, gyrB,* and *glnII* and symbiotic (*nifH* and *nodC*) gene sequences closely aligned the test isolates to the type strains of *Rhizobium muluonense*, *R. paranaense*, *R. pusense, R. phaseoli* and *R. etli.* Subjecting the isolates in this study to further description can potentially reveal novel species. Most of the isolates tested were efficient in fixing nitrogen and elicited greater stomatal conductance and photosynthetic rates in the common bean. Relative effectiveness (RE) varied from 18 to 433%, with 75 (85%) out of the 88 tested isolates being more effective than the nitrate fed control plants.

## Introduction

Common bean (*Phaseolus vulgaris* L.) is an important source of protein to millions of people around the world^1,2^. In low-income farming systems, legumes such as common bean are grown as secondary crops after staples like maize. However, most of these legumes are usually grown under adverse growing conditions with limited water and poor soil fertility, all of which negatively affect their growth and yield^3,4^. To increase yields, synthetic N fertilizers usually offer immediate solution to soil N deficiency^5^. Owing to the high cost and negative environmental footprints of these synthetic fertilizers^6,7^, it has become necessary to explore safer and sustainable biological means to meet legume N demand by identifying indigenous soil microsymbionts that can improve the efficiency of the legume-rhizobium symbiosis.

Even though common bean can fix N_2_ through their symbiotic relationship with rhizobia, the crop is known to derive relatively lower proportion of its N requirement from symbiosis when compared to other legumes^8^. Aside from screening the crop’s diverse germplasm for N_2_ fixation, bioprospecting its microsymbionts can also lead to the identification of strains that can be used to boost this important biological process. Common bean can be nodulated by different rhizobial species from the genera *Rhizobium, Ensifer, and Burkholderia*, and is therefore considered to be a promiscuous host^9^. Because the rhizobial symbionts of legumes have been shown to exhibit some level of biogeographic distribution^10^, it is important to explore their phylogenetic diversity across different environments to unravel potential new species. Despite the importance of common bean in diets and in the Swati (of Eswatini) cropping systems, no information is readily available on the phylogenetic positions of the crop’s native symbionts in the country relative to known reference strains. In this study, we explored the genetic diversity of rhizobia isolated from root nodules of different common bean genotypes grown in the field at the Malkerns Research station in Eswatini using ERIC PCR fingerprinting. The study further assessed phylogenetic relationships between the native rhizobia and reference strains reported from various parts of the globe using the sequence analysis of the 16S rRNA, symbiotic genes (*nifH* and *nodC*) and housekeeping genes (*gln*II, *rpo*B, *dna*K and *gyr*B). In addition, the symbiotic effectiveness of the rhizobial isolates was assessed under glasshouse conditions.

## Results

### Morphophysiological characteristics of rhizobial isolates

A total of 162 bacterial isolates were obtained from root nodules of common bean collected from Malkerns in Eswatini. Of this number, 129 isolates were able to form nodules on the common bean host (Kranskop) in an authentication study in the glasshouse. From the 129 rhizobial isolates, 88 elicited effective nodules while 41 formed ineffective nodules on the host. As expected, there were no nodules on the roots of the control (uninoculated) plants as well as the nitrate fed seedlings.

The effective rhizobial isolates were phenotypically different in terms of colony appearance, shape, size, colour, opacity and texture (Table 1). For example, 60 isolates were fast growers (taking 1–3 days to appear on YMA plates), 27 were intermediate (between 4 and 8 days) and one isolate exhibited slow growth rate and took 10 days to appear on YMA plate. Moreover, 35 of the total isolates had small colony sizes of ≤ 2 mm diameter while 53 isolates had colony diameter between 3 and 7 mm. The colonies were either opaque or translucent in appearance. A greater proportion of the isolates were opaque (68%), having a gummy texture (94%), white in appearance (67%) and flat in shape (69%) (Table 1).

**Table 1:** Isolates used in the diversity study, their host genotypes, and morphological characteristics.

Treatment	Genotype	Growth (days)	Size (mm)	Appearance	Colour	Shape	Texture
TUTPvES 1(1)	DAB 410	5	3	Translucent	Watery	Flat	Gummy
TUTPvES 2(1)	DAB 369	4	4	Opaque	White	Flat	Gummy
TUTPvES 2(2)	DAB 369	2	4	Opaque	White	Dome	Gummy
TUTPvES 2(3)	DAB 369	2	3	Opaque	White	Dome	Gummy
TUTPvES 4(1)	DAB 381	3	2	Opaque	White	Dome	Gummy
TUTPvES 4(2)	DAB 381	3	2	Opaque	White	Dome	Gummy
TUTPvES 4(3)	DAB 381	3	4	Opaque	White	Dome	Gummy
TUTPvES 4(4)	DAB 381	3	5	Translucent	Watery	Flat	Gummy
TUTPvES 5(1)	DAB 447	3	1	Translucent	Watery	Flat	Gummy
TUTPvES 5(2)	DAB 447	4	3	Opaque	White	Flat	Gummy
TUTPvES 5(3)	DAB 447	6	1	Opaque	White	Flat	Gummy
TUTPvES 6(1)	DAB 363	3	4	Opaque	Watery	Flat	Gummy
TUTPvES 7(1)	DAB 429	4	4	Opaque	White	Flat	Gummy
TUTPvES 7(2)	DAB 429	3	5	Translucent	Watery	Flat	Gummy
TUTPvES 7(3)	DAB 429	5	3	Opaque	White	Flat	Gummy
TUTPvES 9(2)	DAB 387	3	2	Opaque	White	Flat	Dry
TUTPvES 10(1)	DAB 386	3	2	Opaque	White	Flat	Gummy
TUTPvES 10(2)	DAB 386	2	3	Opaque	White	Flat	Gummy
TUTPvES 10(4)	DAB 386	3	4	Opaque	White	Flat	Gummy
TUTPvES 11(2)	DAB 470	4	2	Opaque	White	Dome	Gummy
TUTPvES 11(3)	DAB 470	4	4	Translucent	Watery	Dome	Gummy
TUTPvES 12(1)	CIM-SUG-05-01-02	3	2	Opaque	White	Flat	Dry
TUTPvES 13(1)	NUC 461	4	3	Opaque	White	Flat	Gummy
TUTPvES 13(3)	NUC 461	3	2	Translucent	Watery	Flat	Gummy
TUTPvES 14(1)	NUC 451	3	2	Opaque	White	Flat	Gummy
TUTPvES 15(1)	NUC 134	4	2	Opaque	White	Flat	Gummy
TUTPvES 15(2)	NUC 134	4	2	Opaque	White	Flat	Gummy
TUTPvES 16(1)	MCA 78	5	3	Opaque	White	Flat	Dry
TUTPvES 20(1)	NUA 708	3	3	Opaque	White	Dome	Gummy
TUTPvES 21(1)	NUA 735	3	2	Opaque	White	Flat	Gummy
TUTPvES 22(1)	NUA 705	2	5	Translucent	Watery	Flat	Gummy
TUTPvES 23(1)	NUA 730	3	3	Opaque	White	Flat	Dry
TUTPvES 24(1)	ROBA 1	3	5	Translucent	Watery	Flat	Gummy
TUTPvES 25(1)	CIM-KHAK02-53-1	3	< 1	Translucent	Watery	Flat	Gummy
TUTPvES 26(1)	CIM-KHAK02-14-1	3	1	Opaque	White	Flat	Gummy
TUTPvES 27(1)	SER 129	4	2	Opaque	White	Dome	Gummy
TUTPvES 27(2)	SER 129	5	1	Opaque	White	Dome	Gummy
TUTPvES 28(1)	DAB 207	4	2	Opaque	White	Flat	Gummy
TUTPvES 30(1)	DAB 155	4	2	Opaque	White	Flat	Gummy
TUTPvES 30(2)	DAB 155	4	3	Opaque	White	Flat	Gummy
TUTPvES 30(3)	DAB 155	3	5	Translucent	Watery	Flat	Gummy
TUTPvES 30(4)	DAB 155	5	3	Opaque	White	Flat	Gummy
TUTPvES 30(5)	DAB 155	4	3	Opaque	White	Flat	Gummy
TUTPvES 31(1)	CIM-RMO2-76-4	3	4	Translucent	Watery	Flat	Gummy
TUTPvES 31(2)	CIM-RMO2-76-4	4	1	Opaque	White	Flat	Gummy
TUTPvES 32(1)	CIM-KHAK02-20-1	3	2	Opaque	White	Flat	Gummy
TUTPvES 33(1)	CIM-RK06-ALS-S1-2	2	1	Opaque	White	Flat	Gummy
TUTPvES 33(2)	CIM-RK06-ALS-S1-2	3	1	Opaque	White	Dome	Gummy
TUTPvES 34(1)	KAB 77 F7.2-52	10	3	Translucent	Watery	Dome	Gummy
TUTPvES 34(2)	KAB 77 F7.2-52	8	2	Translucent	Watery	Dome	Gummy
TUTPvES 34(3)	KAB 77 F7.2-52	8	3	Translucent	Watery	Flat	Gummy
TUTPvES 36(1)	CIM-RMO5-ALS-103	4	3	Opaque	White	Flat	Gummy
TUTPvES 36(2)	CIM-RMO5-ALS-103	4	2	Opaque	White	Flat	Gummy
TUTPvES 37(1)	CIM-RM00-118-1	3	1	Translucent	Watery	Dome	Gummy
TUTPvES 37(2)	CIM-RM00-118-1	2	3	Opaque	White	Dome	Gummy
TUTPvES 37(3)	CIM-RM00-118-1	3	< 1	Opaque	White	Dome	Gummy
TUTPvES 37(4)	CIM-RM00-118-1	2	7	Translucent	Watery	Dome	Gummy
TUTPvES 38(1)	G 5207	2	3	Opaque	White	Flat	Gummy
TUTPvES 38(2)	G 5207	3	4	Opaque	White	Dome	Gummy
TUTPvES 39(1)	SMC 17	3	2	Opaque	White	Dome	Gummy
TUTPvES 39(3)	SMC 17	5	< 1	Opaque	White	Flat	Dry
TUTPvES 39(4)	SMC 17	2	3	Opaque	White	Flat	Gummy
TUTPvES 40(1)	KAB06F8.8-35	2	6	Opaque	White	Dome	Gummy
TUTPvES 41(2)	KG 98-18	2	4	Opaque	White	Flat	Gummy
TUTPvES 41(3)	KG 98-18	2	3	Translucent	Watery	Flat	Gummy
TUTPvES 42(2)	CIM-RMO2-79-1	2	2	Opaque	White	Flat	Gummy
TUTPvES 42(3)	CIM-RMO2-79-1	2	1	Opaque	White	Flat	Gummy
TUTPvES 43(2)	CIM-RM02-73-1	2	5	Opaque	White	Flat	Gummy
TUTPvES 44(1)	CIM-RM00-141	2	1	Opaque	White	Flat	Gummy
TUTPvES 44(2)	CIM-RM00-141	2	3	Translucent	Watery	Flat	Gummy
TUTPvES 45(1)	CIM-RM00-27-4	3	3	Opaque	White	Flat	Gummy
TUTPvES 47(2)	AND 277	2	3	Opaque	White	Flat	Gummy
TUTPvES 48(1)	KAB 10F2.8-84	4	3	Translucent	Watery	Flat	Gummy
TUTPvES 49(1)	KG 98-18	3	6	Translucent	Watery	Flat	Gummy
TUTPvES 49(2)	KG 98-18	3	2	Translucent	Watery	Dome	Gummy
TUTPvES 53(2)	CIM-RMO1-92-3	2	4	Translucent	Watery	Dome	Gummy
TUTPvES 53(3)	CIM-RMO1-92-3	3	5	Translucent	Watery	Dome	Gummy
TUTPvES 53(4)	CIM-RMO1-92-3	2	4	Translucent	Watery	Dome	Gummy
TUTPvES 54(3)	CIM-RM-03-03-45	3	1	Opaque	White	Flat	Gummy
TUTPvES 55(1)	NAIN DEKYONDO	2	5	Opaque	Watery	Dome	Gummy
TUTPvES 56(1)	MAZ 188	2	3	Translucent	Watery	Flat	Gummy
TUTPvES 57(1)	DAB 559	2	7	Translucent	Watery	Flat	Gummy
TUTPvES 57(2)	DAB 559	2	3	Opaque	White	Flat	Gummy
TUTPvES 57(3)	DAB 559	2	6	Opaque	White	Flat	Gummy
TUTPvES 59(1)	NUA 721	3	2	Opaque	White	Dome	Gummy
TUTPvES 62(2)	CIM-RM-03-27-01	4	3	Translucent	White	Dome	Gummy
TUTPvES 63(1)	NUA 706	2	3	Translucent	Watery	Flat	Gummy
TUTPvES 64(1)	SUGAR 131	3	4	Opaque	White	Flat	Gummy

### ERIC-PCR fingerprints of common bean isolates

**Figure 1:**
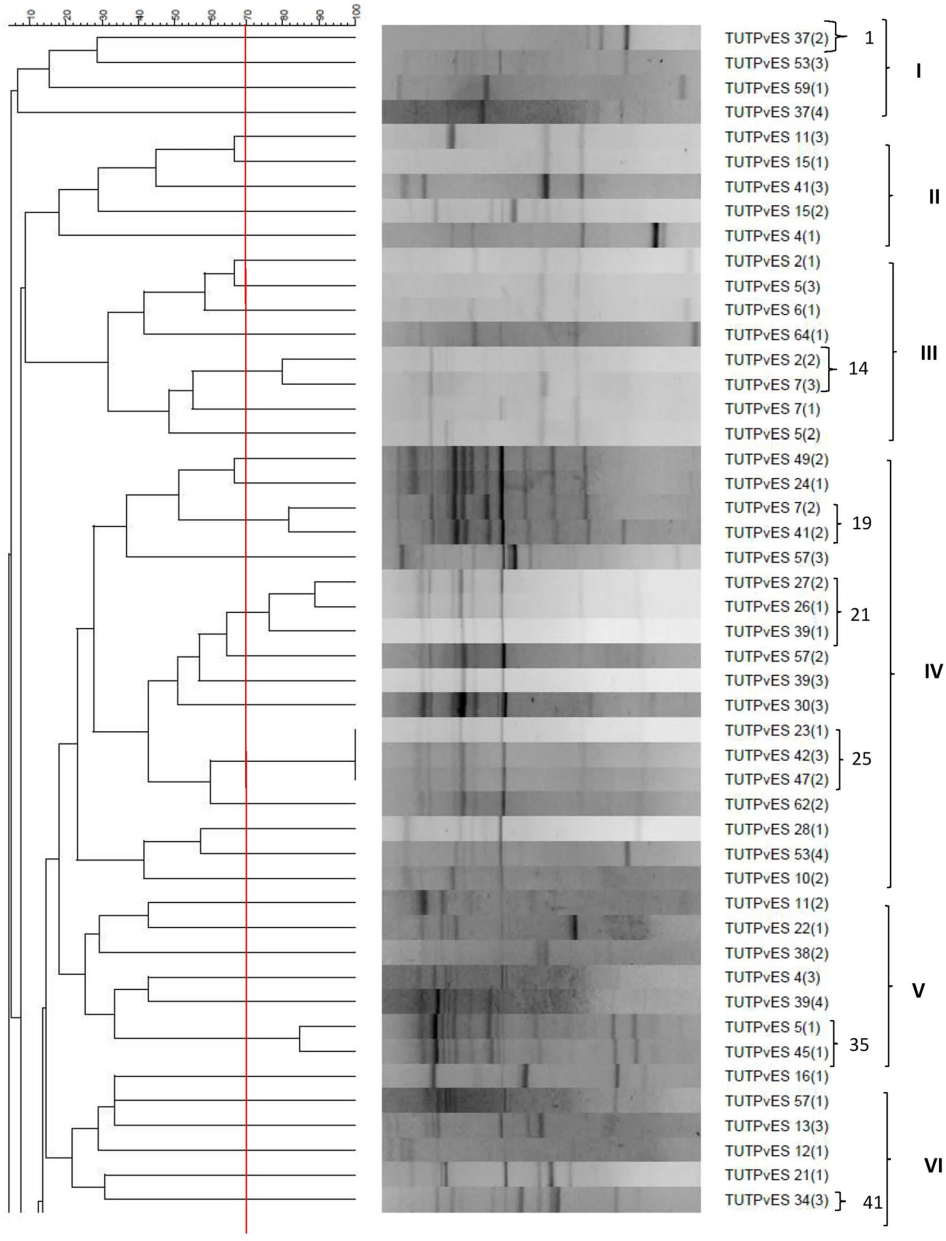

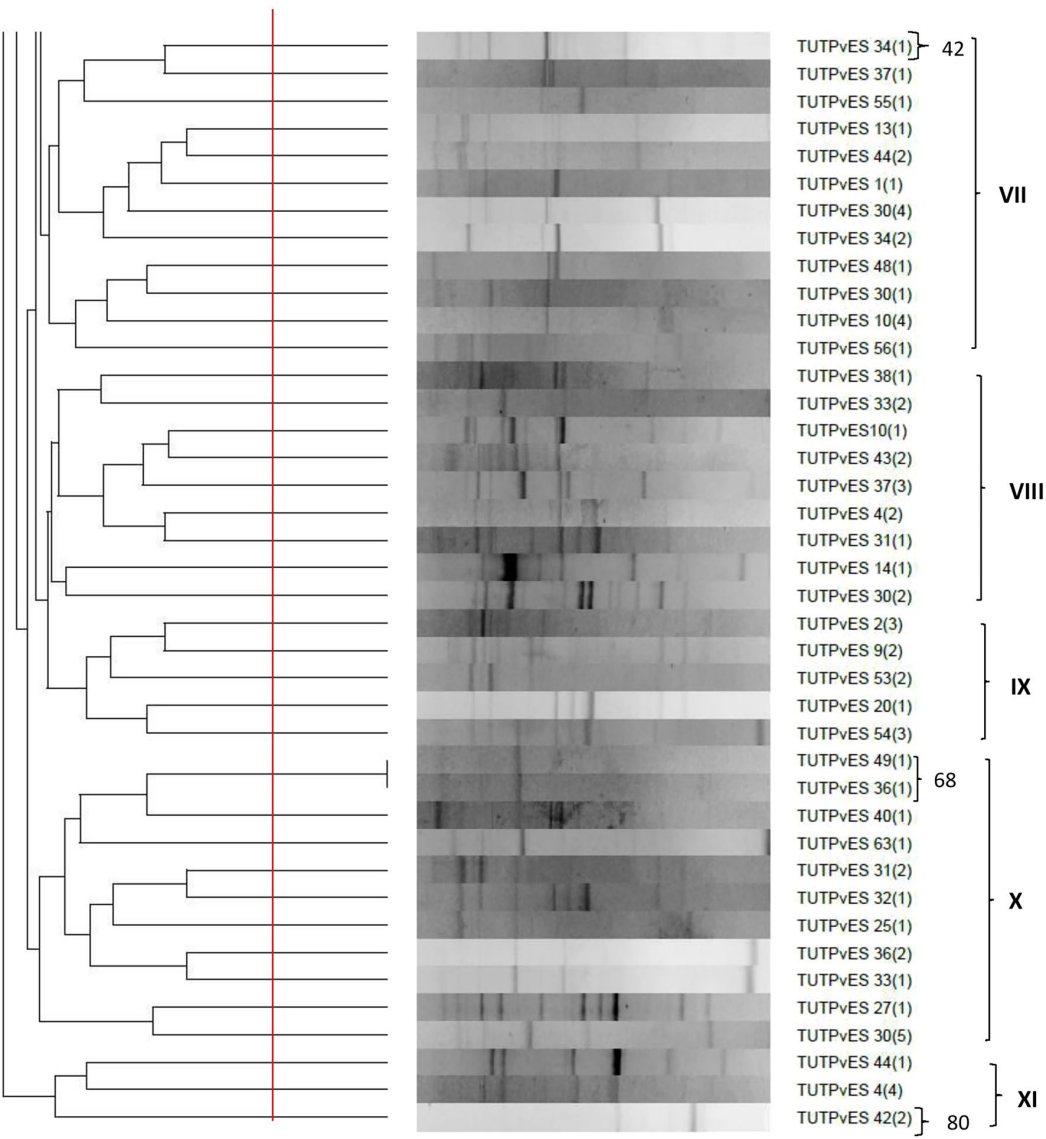
Dendrogram of ERIC fingerprints revealing the presence of high genetic diversity among the 129 indigenous rhizobial microsymbionts of common bean from Malkerns in Eswatini. Red vertical line indicates 70% similarity cut of point.

The ERIC-PCR profiles grouped the isolates of common bean into eleven (11) major clusters if considered at 20% similarity level. However, these isolates represented 80 ERIC-PCR types when considered at a 70% similarity level (Fig. 1). In terms of the number of isolates, Cluster IV was the largest and contained 18 isolates while Cluster XI contained the least number of isolates, namely, TUTPvES 44(1), TUTPvES 4(4) and TUTPvES 42(2) (Fig. 1). Some clusters grouped isolates from different common bean genotypes; for example, Cluster II grouped isolates TUTPvES 11(3), TUTPvES 15(1), TUTPvES 41(3), TUTPvES (2) and TUTPvES 4(1) which were isolated from the root nodules of genotypes DAB 470, NUC 134, KG 98-36, NUC 134 and DAB 387, respectively (Fig. 1; Table 1).

### Phylogeny of isolates based on 16S rRNA gene sequences

**Figure 2:**
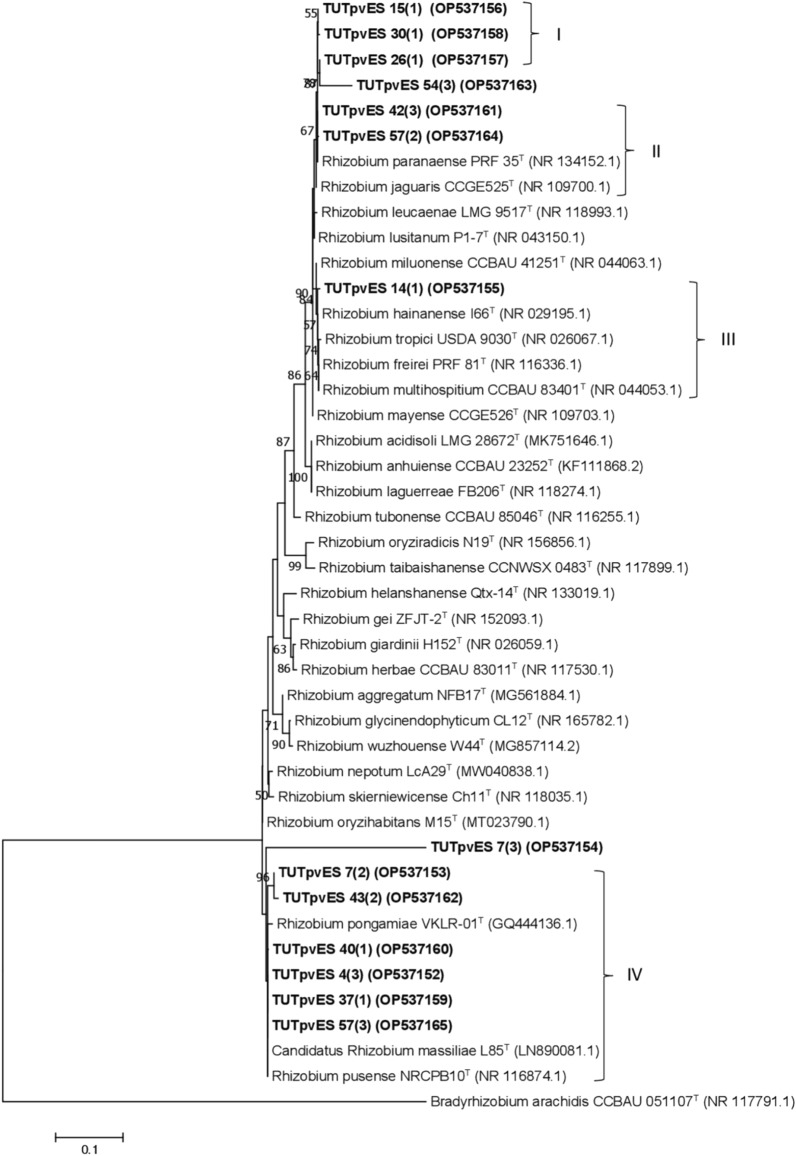
Maximum-likelihood phylogenetic tree inferred from 16S rRNA gene sequences of common bean symbionts from Eswatini. Phylogenetic trees were inferred using MEGA 7 software 35. The Kimura 2-paramete model with uniform rates among the sites was used to calculate evolutionary distances.

The 16S rRNA phylogeny grouped a total of 14 isolates into four clusters (Cluster I, II, III and IV) which were closely related to known *Rhizobium* type strains, except for isolate TUTPvES 7(3) which stood separately from any of the type strains (Fig. 2). In Cluster I, isolates TUTPvES 15(1), TUTPvES 26(1) and TUTPvES 30(1) (with 99.4–99.7% sequence similarities) shared 99.5–99.9% sequence similarity with the type strains *R. paranaense* and *R. jaguaris*. Isolate TUTPvES 54(3) formed an outgroup of isolates in Cluster I. Furthermore, isolates TUTPvES 42(3) and TUTPvES 57(2) (with 99.9% sequence similarity) respectively shared 99.7% and 100% sequence similarity with *R. jaguaris* and *R. paranaense* in Cluster II (Fig. 2). Isolate TUTPvES 14(1) shared 99.4% to 99.5% sequence similarity with *R. freirei, R. hainanense, R. multihospitum* and *R. tropici* with 95.5% bootstrap support in Cluster III (Fig. 2). Moreover, isolates TUTPvES 4(3), TUTPvES 7(2), TUTPvES 37(1), TUTPvES 40(1), TUTPvES 43(2) and TUTPvES 57(3) grouped together in Cluster IV with 98.1–100% sequence similarity; within this Cluster IV, isolates TUTPvES 4(3) and TUTPvES 57(3) were identical to the type strain *Candidatus Rhizobium massiliae* while the remaining isolates shared 98.1–99.9% sequence similarities with *R. pusense* and *R. pongamiae* (Fig. 2).

### Phylogenies based on housekeeping genes (*dnaK, glnII, gyrB* and *rpoB*)

Four housekeeping genes (*dnaK, glnII, gyrB* and *rpoB)* were selected for a robust multilocus sequence phylogenetic analysis. The PCR amplification of the *dnaK*, *glnII*, *rpoB*, and *gyrB* genes yielded bands of about 650, 700, 700 and 500 bp, respectively for the selected rhizobial population. The primers used and temperature conditions are listed in supplementary Table S3. The maximum likelihood phylogenetic trees based on the individual gene sequences placed the isolates in different clusters within the genus *Rhizobium*. Some isolates consistently grouped together in the 16S rRNA phylogram as well as in the phylograms based on individual housekeeping genes, while others showed discrepancies. For example, isolates such as TUTPvES 7(2) and TUTPvES 43(2) which grouped with other isolates in Cluster IV in the 16S rRNA gene phylogeny showed a high divergence from those isolates in the *dnaK, glnII, gyrB and rpoB* phylogenies (Fig. 2; Supplementary Fig. S1–S4). Nevertheless, the grouping of isolates in the individual housekeeping genes were largely consistent. For example, isolate TUTPvES 14(1) aligned with *R. tropici* in the *glnII*, *gyrB* and *rpoB* phylogenies, but stood separately in the *dnaK* phylogeney due to the absence of the type strain in that phylogram (Supplementary Fig. S1–S4).

### Phylogeny based on concatenated gene sequences

**Figure 3:**
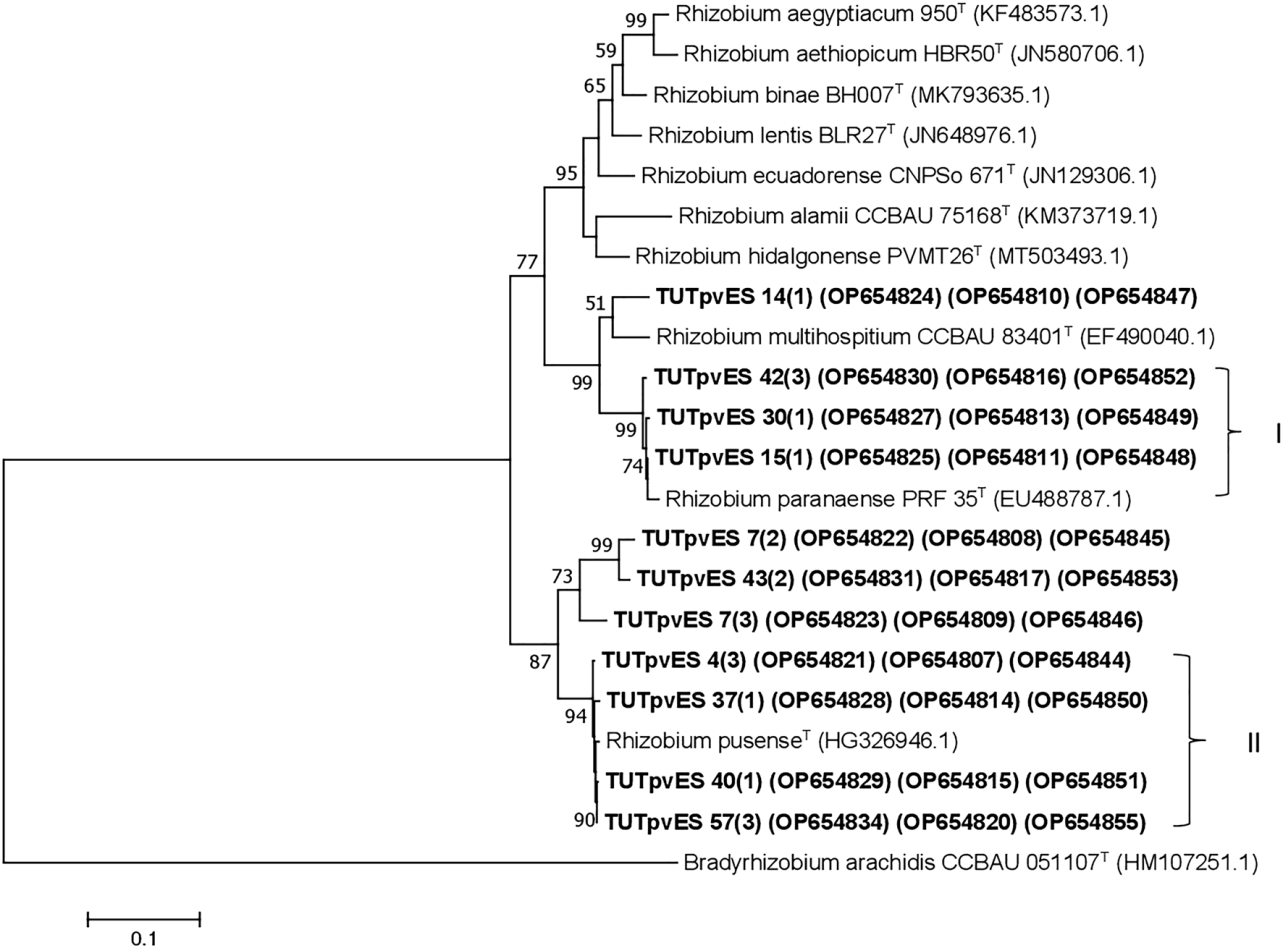
Maximum-likelihood phylogeny of microsymbionts of common bean from Eswatini inferred from concatenated *glnII* + *dnaK* + *rpoB* gene sequences. Phylogenetic trees were inferred using MEGA 7 software 35. The Kimura 2-paramete model with uniform rates among the sites was used to calculate evolutionary distances.

A concatenated gene phylogeny based on the combined sequences of *glnII* + *dnaK* + *rpoB* grouped a total of eleven (11) isolates into two main clusters (Cluster I and II) (Fig. 3). In Cluster I, isolates TUTPvES 15(1), TUTPvES 30(1) and TUTPvES 42(3) (with 99.2–99.6% sequence similarity) shared 98.3–98.9% sequence similarity with *R. paranaense* with 99% bootstrap support (Fig. 3). On the other hand, isolate TUTPvES 14(1) was highly divergent from the reference type strains and shared only 93.2% sequence similarity with *R. multihospitium*, the closest related type strain. Similarly, isolates TUTPvES 7(2) and TUTPvES 43(2) stood separately and shared only 91.5% and 92.4% sequence similarity with *R. pusense* and *R. paranaense*, respectively, the closest related type strains (Fig. 3). In Cluster III, isolates TUTPvES 37(1), TUTPvES 40 (1) and TUTPvES 57(3) (with 99.2–99.8% sequence similarity) aligned with *R. pusense* with 99.1–99.5% sequence similarity (Fig. 3).

### Phylogeny based on symbiotic gene sequences (*nodC* and *nifH*)

**Figure 4:**
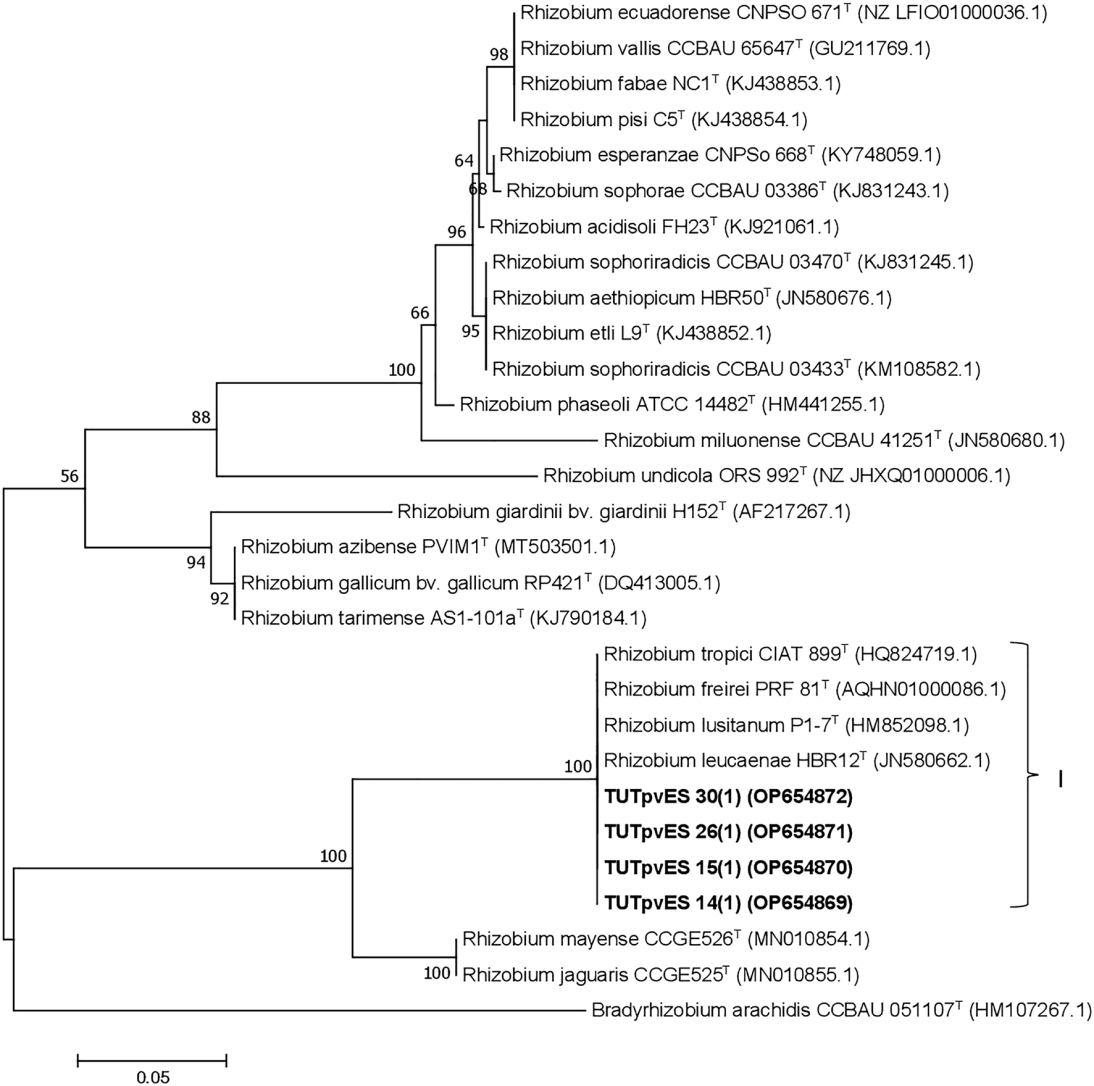
Maximum-likelihood phylogeny of microsymbionts of common bean from Eswatini inferred from *nodC* gene sequences. Phylogenetic trees were inferred using MEGA 7 software 35. The Kimura 2-paramete model with uniform rates among the sites was used to calculate evolutionary distances.

**Figure 5:**
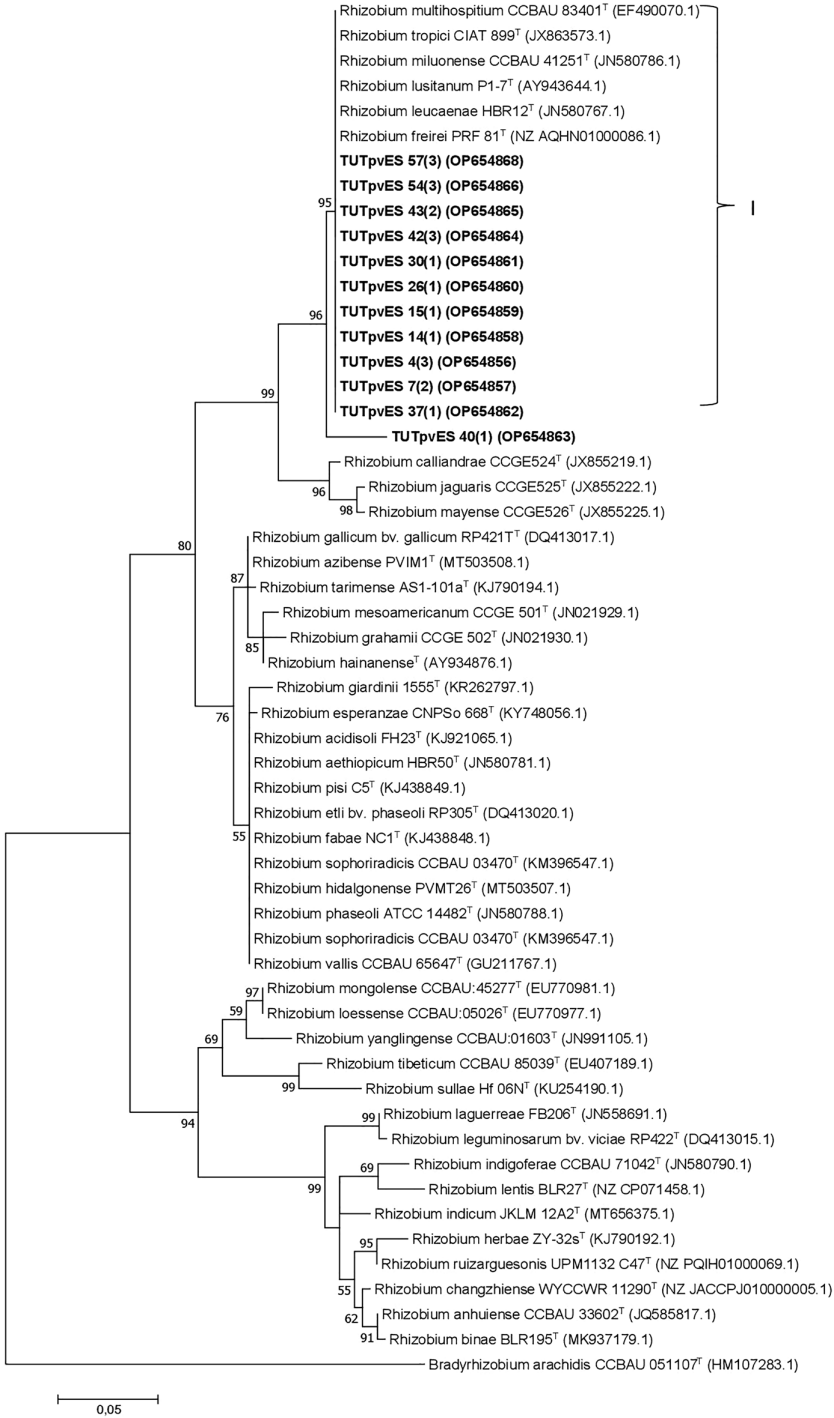
Maximum-likelihood phylogeny of microsymbionts of common bean from Eswatini inferred from *nifH* gene sequences. Phylogenetic trees were inferred using MEGA 7 software 35. The Kimura 2-paramete model with uniform rates among the sites was used to calculate evolutionary distances.

The phylogeny based on *nodC* gene grouped all four isolates, TUTPvES 30(1), TUTPvES 26(1), TUTPvES 15(1) and TUTPvES 14(1) with *R. tropici, R. freirei, R. lusitanum and R. leucaenae* with 100% similarity (Fig. 4). The phylogeny based on the *nifH* gene also grouped 12 identical isolates with the type strains *R. tropici, R. freirei, R. leucaenae, R. lusitanum, R. miluonense and R. multihospitium* with 100% sequence similarity and 95% bootstrap support; however, isolate TUTPvES 40(1) formed an out group of the Cluster and shared only 96.6% sequence similarity with the type strains (Fig. 5).

### Nodulation and plant growth induced by isolates

The results showed significant differences (*p* ≤ 0.05) in the nodule number, nodule weight and shoot dry matter (SDM) of common beans inoculated with the different rhizobial isolates (Table 2). Isolates TUTPvES 34(2), TUTPvES 20(1), TUTPvES 2(3) and TUTPvES 22(1) had significantly higher nodule numbers than the remaining isolates, recording 756, 750, 646 and 537 nodules per plant, respectively, whereas isolates TUTPvES 48(1), TUTPvES 13(3), TUTPvES 34(1) and TUTPvES 7(1) recorded the least nodule numbers, with values of 5, 20, 22, 31 nodules per plant, respectively. Isolates TUTPvES 34(2), TUTPvES 20(1) and TUTPvES 6(1) coupled higher nodule number with high nodule weight; however, isolates TUTPvES 62(2) and TUTPvES 43(2) had lower nodule number but elicited greater nodule dry matter (Table 2).

**Table 2:** Plant growth, nodulation and photosynthesis parameter of common bean inoculated with indigenous rhizobial strains in the glasshouse.

Isolates	Shoot	Nodule number	Nodule dry weight	A	Gs	E	RE
	g. plant^-1^	plant^-1^	g. plant^-1^	µmol (Co_2_) m^-2^ s^-1^	mol (H_2_O) m^-2^ s^-1^	mol (H_2_O) m^-2^ s^-1^	%
TUTPvES 1(1)	2.40 ± 0.06 m–s	336 ± 5.04i	0.328 ± 0.01 h–k	5.233 ± 0.31t–w	0.14 ± 0.01w–D	4.13 ± 0.01t–z	180 ± 4 m–r
TUTPvES 2(1)	0.83 ± 0.12D–J	134 ± 6.69u–x	0.229 ± 0.07 m–u	7.091 ± 0.07st	0.12 ± 0.01y–f	3.09 ± 0.19z–E	63 ± 9B–G
TUTPvES 2(2)	2.67 ± 0.17j–q	129 ± 4.33v–A	0.234 ± 0.01 m–t	19.584 ± 1.34a–f	0.34 ± 0.05c–i	7.05 ± 0.81b–j	200 ± 13j–p
TUTPvES 2(3)	4.60 ± 0.12b	646 ± 3.18b	0.416 ± 0.01de	20.614 ± 0.69a	0.29 ± 0.03 h–o	6.67 ± 0.46d–l	345 ± 9b
TUTPvES 4(1)	0.97 ± 0.09B–H	100 ± 0.88C–F	0.229 ± 0.02 m–u	2.563 ± 0.00x–C	0.11 ± 0.03z–G	3.36 ± 0.09x–D	73 ± 7A–F
TUTPvES 4(2)	1.53 ± 0.15v–B	153 ± 5.77st	0.306 ± 0.01kl	1.077 ± 0.15ABC	0.03 ± 0.00GH	1.12 ± 0.05GHI	115 ± 11u–A
TUTPvES 4(3)	2.80 ± 0.12 s–w	156 ± 3.46st	0.055 ± 0.01GHI	18.679 ± 0.33a–i	0.24 ± 0.03 m–u	5.19 ± 0.07 m–u	210 ± 9i–o
TUTPvES 4(4)	3.77 ± 0.15cde	275 ± 3.76 k	0.353 ± 0.01 g–j	17.178 ± 0.83f.–l	0.33 ± 0.01d–k	6.40 ± 0.29e–o	283 ± 11cde
TUTPvES 5(1)	1.25 ± 0.14x–F	56 ± 3.06JK	0.234 ± 0.01 m–t	20.601 ± 1.10a	0.40 ± 0.03a–e	7.79 ± 0.47a–e	94 ± 11w–D
TUTPvES 5(2)	3.47 ± 0.21d–h	370 ± 11.26 h	0.258 ± 0.02 mn	19.154 ± 0.95a–h	0.30 ± 0.02 h–o	6.69 ± 0.07d–l	260 ± 15d–h
TUTPvES 5(3)	3.17 ± 0.17f.–k	259 ± 3.46kl	0.257 ± 0.03 mn	19.689 ± 0.45a–f	0.32 ± 0.03f.–m	6.84 ± 0.59c–k	238 ± 13f.–k
TUTPvES 6(1)	2.80 ± 0.17i–p	500 ± 6.06e	0.629 ± 0.01a	17.993 ± 0.06b–j	0.449 ± 0.04a	8,335 ± 0.07	210 ± 13i–o
TUTPvES 7(1)	1.17 ± 0.17z–F	31 ± 2.60L	0.067 ± 0.00FGH	3.417 ± 0.01v–A	0.08 ± 0.00B–H	2.89 ± 0.00z–F	88 ± 13y–D
TUTPvES 7(2)	0.37 ± 0.12IJK	53 ± 3.18JK	0.102 ± 0.01EF	2.366 ± 0.00x–C	0.07 ± 0.00C–H	2.48 ± 0.01B–G	28 ± 9GH
TUTPvES 7(3)	1.27 ± 0.15x–F	106 ± 3.46B–F	0.203 ± 0.01p–x	0.725 ± 0.05BC	0.03 ± 0.00GH	1.09 ± 0.01HI	95 ± 11w–D
TUTPvES 9(2)	3.67 ± 0.09d–g	431 ± 4.91 g	0.446 ± 0.03 cd	16.991 ± 0.36 g–m	0.36 ± 0.00b–i	7.78 ± 0.168a–e	275 ± 7d–g
TUTPvES 10(1)	2.17 ± 0.33o–u	208 ± 4.62op	0.242 ± 0.01 m–s	17.742 ± 1.30d–k	0.37 ± 0.05a–h	6.88 ± 0.59c–k	163 ± 25n–t
TUTPvES 10(2)	1.07 ± 0.09B–F	145 ± 5.70tuv	0.199 ± 0.04p–y	4.280 ± 0.02u–x	0.17 ± 0.00u–A	3.95 ± 0.09u–A	80 ± 6.6A–D
TUTPvES 10(4)	2.70 ± 0.17i–p	223 ± 2.02no	0.180 ± 0.00v–B	20.360 ± 1.02abc	0.41 ± 0.05a–d	7.12 ± 0.48b–i	203 ± 13i–p
TUTPvES 11(2)	1.37 ± 0.09w–E	89 ± 5.20FGH	0.361 ± 0.02ghi	0.699 ± 0.02BC	0.14 ± 0.04w–E	3.13 ± 0.91y–E	103 ± 7v–C
TUTPvES 11(3)	1.27 ± 0.15x–F	170 ± 11.25rs	0.069 ± 0.01FGH	20.447 ± 1.11ab	0.42 ± 0.02ab	7.11 ± 0.02b–i	95 ± 11w–D
TUTPvES 12(1)	2.27 ± 0.15n–u	133 ± 4.33u–y	0.188 ± 0.01t–z	3.024 ± 0.81w–C	0.07 ± 0.01C–H	1.77 ± 0.06E–I	170 ± 11n–t
TUTPvES 13(1)	0.40 ± 0.10G–K	517 ± 2.03d	0.367 ± 0.02fgh	9.850 ± 0.08pqr	0.19 ± 0.01t–z	4.43 ± 0.23 s–y	30 ± 8E–H
TUTPvES 13(3)	0.43 ± 0.07G–K	20 ± 1.15LM	0.032 ± 0.01HIJ	15.976 ± 0.26j–m	0.21 ± 0.02p–w	4.65 ± 0.36r–x	33 ± 5E–H
TUTPvES 14(1)	2.67 ± 0.09j–q	336 ± 9.24i	0.149 ± 0.03z–D	15.077 ± 0.27 lm	0.27 ± 0.01j–r	5.08 ± 0.37o–v	200 ± 7j–p
TUTPvES 15(1)	2.00 ± 0.50r–y	67 ± 8.82IJK	0.149 ± 0.01z–D	17.945 ± 0.64c–j	0.31 ± 0.02 h–o	6.36 ± 0.33f.–p	150 ± 38q–u
TUTPvES 15(2)	1.17 ± 0.17z–F	139 ± 4.04t–w	0.013 ± 0.00IJ	2.535 ± 0.83x–C	0.05 ± 0.02FGH	1.50 ± 0.61F–I	88 ± 13y–D
TUTPvES 16(1)	1.37 ± 0.09w–E	76 ± 2.31GHI	0.014 ± 0.00IJ	16.292 ± 0.01i–m	0.33 ± 0.00d–k	7.41 ± 0.01b–g	103 ± 7v–C
TUTPvES 20(1)	5.77 ± 0.32a	750 ± 19.15a	0.507 ± 0.00b	20.212 ± 0.28a–d	0.37 ± 0.03a–h	7.47 ± 0.11b–g	433 ± 24a
TUTPvES 21(1)	1.70 ± 0.12u–B	71 ± 3.18HIJ	0.061 ± 0.01FGH	1.385 ± 0.07y–C	0.06 ± 0.00E–H	2.05 ± 0.04D–H	128 ± 9t–z
TUTPvES 22(1)	2.33 ± 0.09n–t	537 ± 9.53c	0.303 ± 0.01kl	18.907 ± 1.15a–h	0.23 ± 0.02n–u	5,561 ± 0.19 k–s	175 ± 7n–s
TUTPvES 23(1)	2.80 ± 0.15i–p	450 ± 5.77f.	0.212 ± 0.01n–w	18.552 ± 0.59a–i	0.31 ± 0.04f.–n	6.40 ± 0.78e–o	210 ± 11i–o
TUTPvES 24(1)	4.67 ± 0.23b	370 ± 11.26 h	0.200 ± 0.01p–y	20.716 ± 0.75a	0.29 ± 0.03 h–p	6.31 ± 0.51f.–p	350 ± 18b
TUTPvES 25(1)	3.10 ± 0.06 g–l	234 ± 1.20 mn	0.265 ± 0.01 l–m	18.457 ± 0.59a–i	0.32 ± 0.01f.–l	6.99 ± 0.19b–j	233 ± 4 g–l
TUTPvES 26(1)	3.30 ± 0.06e–i	338 ± 4.10i	0.308 ± 0.01jk	16.292 ± 0.00i–m	0.33 ± 0.00d–k	7.41 ± 0.01b–g	248 ± 4e–i
TUTPvES 27(1)	1.40 ± 0.06w–D	87 ± 1.73FGH	0.208 ± 0.00o–w	7.041 ± 0.01st	0.16 ± 0.00v–B	4,792 ± 0.04	105 ± 4v–B
TUTPvES 27(2)	2.97 ± 0.09 h–m	335 ± 8.66i	0.319 ± 0.00ijk	9.448 ± 0.01qr	025 ± 0.00 l–u	5.43 ± 0.09 l–t	223 ± 7 h–m
TUTPvES 28(1)	3.00 ± 0.29 h–m	88 ± 1.45FGH	0.224 ± 0.01 m–v	3.696 ± 0.58v–z	0.05 ± 0.00FGH	1.89 ± 0.09E–I	225 ± 22 h–m
TUTPvES 30(1)	2.77 ± 0.43i–p	140 ± 5.77t–w	0.117 ± 0.01DE	8.892 ± 0.01rs	0.19 ± 0.01 s–y	5.20 ± 0.11 m–u	208 ± 33i–p
TUTPvES 30(2)	4.03 ± 0.12 cd	436 ± 9.24 fg	0.491 ± 0.01b	2.107 ± 0.02x–C	0.02 ± 0.00H	0.66 ± 0.04I	303 ± 9 cd
TUTPvES 30(3)	3.50 ± 0.29d–h	219 ± 5.77no	0.320 ± 0.01ijk	12.741 ± 0.24no	0.18 ± 0.03t–z	5.06 ± 0.61o–v	263 ± 22d–h
TUTPvES 30(4)	3.70 ± 0.17c–f	154 ± 5.49st	0.159 ± 0.02x–D	15.598 ± 0.04j–m	0.25 ± 0.00 k–t	6.42 ± 0.21e–o	278 ± 13c–f
TUTPvES 30(5)	3.77 ± 0.15cde	276 ± 2.60 k	0.231 ± 0.01 m–t	19.461 ± 0.25a–g	0.31 ± 0.06 h–o	6.589 ± 0.87e–l	283 ± 11cde
TUTPvES 31(1)	2.27 ± 0.15n–u	102 ± 2.85C–F	0.237 ± 0.00 m–s	3.786 ± 0.71v–y	0.08 ± 0.02B–H	2.35 ± 0.61C–H	170 ± 11n–t
TUTPvES 31(2)	3.77 ± 0.15cde	294 ± 5.77i	0.352 ± 0.02 g–j	16.707 ± 0.46 h–m	0.32 ± 0.02e–l	6.53 ± 0.45e–n	283 ± 11cde
TUTPvES 32(1)	3.00 ± 0.06 h–m	228 ± 4.06 mn	0.244 ± 0.00 m–r	14.695 ± 0.86 mn	0.27 ± 0.02j–s	6.13 ± 0.41 g–q	225 ± 4 h–m
TUTPvES 33(1)	1.13 ± 0.09A–F	115 ± 3.18y–C	0.220 ± 0.02 m–v	3.422 ± 0.50v–A	0.05 ± 0.00FGH	1.82 ± 0.11E–I	85 ± 7z–D
TUTPvES 33(2)	0.77 ± 0.09E–K	100 ± 4.62C–F	0.244 ± 0.00 m–r	5.505 ± 3.49tuv	0.10 ± 0.07A–G	2,890 ± 1.63	58 ± 7C–H
TUTPvES 34(1)	1.20 ± 0.15x–F	22 ± 1.73L	0.326 ± 0.00 h–k	6.401 ± 1.23tu	0.13 ± 0.03x–F	4.05 ± 0.69u–z	90 ± 11x–D
TUTPvES 34(2)	3.90 ± 0.35 cd	756 ± 15.88a	0.645 ± 0.00a	11.341 ± 0.91opq	0.21 ± 0.01q–x	5.16 ± 0.16o–v	293 ± 26 cd
TUTPvES 34(3)	2.57 ± 0.20j–r	327 ± 15.30i	0.404 ± 0.00def	6.673 ± 0.00t	0.14 ± 0.00w–C	4.62 ± 0.02 s–x	193 ± 15 k–q
TUTPvES 36(1)	0.37 ± 0.09IJK	223 ± 4.33no	0.341 ± 0.02 g–k	17.922 ± 0.88c–j	0.29 ± 0.00i–p	6.67 ± 0.180d–l	28 ± 6GH
TUTPvES 36(2)	2.50 ± 0.17 l–r	141 ± 1.73t–w	0.244 ± 0.00 m–r	9.318 ± 0.89qr	0.17 ± 0.04u–A	4.85 ± 0.83q–w	188 ± 13i–q
TUTPvES 37(1)	1.80 ± 0.17t–y	94 ± 2.60D–G	0.138 ± 0.00A–E	12.591 ± 1.24no	0.19 ± 0.01r–y	5.14 ± 0.41o–v	128 ± 15t–z
TUTPvES 37(2)	2.63 ± 0.13j–q	156 ± 3.76st	0.060 ± 0.00FGH	17.712 ± 0.32e–k	0.24 ± 0.00 l–u	5.75 ± 0.03j–s	198 ± 10j–p
TUTPvES 37(3)	2.27 ± 0.09n–u	262 ± 6.64kl	0.264 ± 0.01 lm	12.734 ± 1.19no	0.20 ± 0.00q–x	5.01 ± 0.10p–v	170 ± 7n–t
TUTPvES 37(4)	1.50 ± 0.173v–C	104 ± 3.18C–F	0.170 ± 0.01w–C	11.757 ± 0.69op	0.23 ± 0.02o–v	5.16 ± 0.42 m–v	113 ± 13u–A
TUTPvES 38(1)	3.00 ± 0.12 h–m	271 ± 10.68 k	0.419 ± 0.02de	15.441 ± 1.87klm	0.31 ± 0.03 g–n	6.77 ± 0.67d–l	225 ± 9 h–l
TUTPvES 38(2)	1.20 ± 0.12x–F	245 ± 3.18 lm	0.252 ± 0.00mno	17.742 ± 0.27d–k	0.30 ± 0.02 h–o	6.59 ± 0.21e–l	90 ± 9x–D
TUTPvES 39(1)	1.90 ± 0.06 s–w	219 ± 10.68no	0.257 ± 0.00 mn	2.852 ± 0.00w–C	0.08 ± 0.00B–H	2.43 ± 0.03C–H	143 ± 4r–v
TUTPvES 39(3)	0.90 ± 0.06C–I	56 ± 1.15JK	0.041 ± 0.00HIJ	2.196 ± 0.02x–C	0.07 ± 0.00C–H	2.10 ± 0.07D–H	65 ± 5B–G
TUTPvES 39(4)	1.90 ± 0.06 s–w	168 ± 7.22rs	0.097 ± 0.01EFG	3.161 ± 0.53v–B	0.14 ± 0.03w–E	3.80 ± 0.88v–B	138 ± 3r–w
TUTPvES 40(1)	2.27 ± 0.09n–u	151 ± 1.73stu	0.038 ± 0.00HIJ	16.707 ± 0.46 h–m	0.32 ± 0.02f.–l	6.53 ± 0.45e–n	170 ± 7n–t
TUTPvES 41(2)	4.27 ± 0.15bc	92 ± 1.73EFG	0.073 ± 0.01FGH	19.313 ± 0.84a–g	0.36 ± 0.01b–i	7.19 ± 0.06b–h	320 ± 11bc
TUTPvES 41(3)	3.67 ± 0.44d–g	67 ± 1.15IJK	0.044 ± 0.01HIJ	19.274 ± 0.40a–g	0.40 ± 0.02a–f	7.36 ± 0.15b–h	275 ± 33d–g
TUTPvES 42(2)	0.87 ± 0.07D–J	72 ± 2.08HIJ	0.055 ± 0.01GHI	3.946 ± 0.07vwx	0.08 ± 0.01B–H	2.60 ± 0.32B–F	65 ± 5B–G
TUTPvES 42(3)	1.53 ± 0.18v–B	131 ± 5.20v–z	0.135 ± 0.08B–E	19.241 ± 0.46a–g	0.39 ± 0.03a–g	7.58 ± 0.42a–f	115 ± 13u–A
TUTPvES 43(2)	3.23 ± 0.15e–k	192 ± 6.65pq	0.478 ± 0.02bc	17.476 ± 0.87e–k	0.27 ± 0.03j–q	5.69 ± 0.26j–s	243 ± 11e–j
TUTPvES 44(1)	1.30 ± 0.12x–E	117 ± 1.73x–C	0.195 ± 0.01 s–y	2.246 ± 0.01x–C	0.06 ± 0.00D–H	1.87 ± 0.11E–I	98 ± 9w–D
TUTPvES 44(2)	1.00 ± 0.06B–H	146 ± 3.46tuv	0.155 ± 0.03y–D	1.908 ± 0.14x–C	0.06 ± 0.00E–H	1.87 ± 0.09E–I	75 ± 4A–F
TUTPvES 45(1)	3.17 ± 0.09f.–k	114 ± 3.46y–C	0.196 ± 0.00p–y	19.687 ± 0.36a–e	0.37 ± 0.01b–i	7.66 ± 0.31a–f	238 ± 7f.–k
TUTPvES 47(2)	1.50 ± 0.29v–C	111 ± 5.51A–E	0.033 ± 0.01HIJ	3.464 ± 0.04v–A	0.07 ± 0.00C–H	1.78 ± 0.07E–I	113 ± 22u–A
TUTPvES 48(1)	0.70 ± 0.12F–K	5 ± 0.88MN	0.002 ± 0.00 J	2.561 ± 0.03x–C	0.07 ± 0.00C–H	2,006 ± 0.07D–I	53 ± 9D–H
TUTPvES 49(1)	1.37 ± 0.09w–E	50 ± 1.15 K	0.100 ± 0.00EFG	2.625 ± 0.02x–C	0.09 ± 0.00B–H	2.68 ± 0.01A–F	103 ± 7v–C
TUTPvES 49(2)	3.67 ± 0.09d–g	246 ± 3.18 lm	0.258 ± 0.02 mn	19.548 ± 0.04a–f	0.45 ± 0.03a	8.02 ± 0.03a–d	275 ± 7d–g
TUTPvES 53(2)	1.77 ± 0.15t–z	94 ± 2.60D–G	0.131 ± 0.01CDE	3.070 ± 0.09w–C	0.05 ± 0.00FGH	2.07 ± 0.01D–H	133 ± 11 s–y
TUTPvES 53(3)	1.80 ± 0.17t–y	113 ± 6.64z–D	0.212 ± 0.01n–w	15.778 ± 0.61j–m	0.25 ± 0.00 l–u	6.00 ± 0.10 h–r	135 ± 13 s–x
TUTPvES 53(4)	0.23 ± 0.03 K	341 ± 5.48i	0.381 ± 0.01efg	2.402 ± 0.44x–C	0.05 ± 20.00FGH	1.74 ± 0.15E–I	18 ± 2.5H
TUTPvES 54(3)	2.77 ± 0.15i–p	71 ± 3.18HIJ	0.061 ± 0.01FGH	17.559 ± 0.81e–k	0.42 ± 0.04abc	7.00 ± 0.47b–j	208 ± 11i–p
TUTPvES 55(1)	4.00 ± 0.29 cd	124 ± 1.20w–B	0.141 ± 0.01A–E	6.951 ± 0.44st	0.15 ± 0.01w–C	4.42 ± 0.29 s–y	300 ± 22 cd
TUTPvES 56(1)	0.97 ± 0.03B–H	237 ± 4.81 mn	0.134 ± 0.01CDE	1.239 ± 0.07z–C	0.05 ± 0.00FGH	1.80 ± 0.13E–I	73 ± 3A–F
TUTPvES 57(1)	2.77 ± 0.15i–p	523 ± 4.62 cd	0.416 ± 0.01de	6.371 ± 0.12tu	0.12 ± 0.00y–F	3.58 ± 0.09w–C	208 ± 11i–p
TUTPvES 57(2)	2.30 ± 0.12n–t	187 ± 1.73q	0.222 ± 0.01 m–v	18.610 ± 1.27a–i	0.37 ± 0.07a–h	7.68 ± 0.91a–f	173 ± 9n–s
TUTPvES 57(3)	2.53 ± 0.29 l–r	111 ± 5.8A–E	0.196 ± 0.00p–y	19.021 ± 0.58a–h	0.37 ± 0.02a–h	8.19 ± 0.08abc	190 ± 22i–q
TUTPvES 59(1)	2.87 ± 0.23i–n	180 ± 6.69qr	0.158 ± 0.02x–D	19.590 ± 0.74a–f	0.44 ± 0.06ab	8.752 ± 0.53	215 ± 18i–o
TUTPvES 62(2)	3.77 ± 0.15cde	333 ± 5.92i	0.644 ± 0.02a	2.434 ± 0.05x–C	0.08 ± 0.00B–H	2.92 ± 0.02z–E	283 ± 11cde
TUTPvES 63(1)	2.17 ± 0.20o–u	166 ± 3.48rs	0.301 ± 0.00kl	1.414 ± 0.03y–C	0.06 ± 0.00E–H	2.21 ± 0.05D–H	163 ± 15n–t
TUTPvES 64(1)	3.23 ± 0.15e–k	145 ± 3.18tuv	0.182 ± 0.01u–A	2.519 ± 0.05x–C	0.07 ± 0.00C–H	2.55 ± 0.03B–F	243 ± 11e–j
Nitrate (5 mM)	1.33 ± 0.18w–E	NA	NA	2.134 ± 0.09x–C	0.08 ± 0.00B–H	2.82 ± 0.00z–F	100 ± 13v–C
Uninoculated	0.30 ± 0.06JK	NA	NA	0.627 ± 0.02C	0.05 ± 0.01FGH	1,88 ± 0.00E–I	23 ± 4GH
F statistics	45.60**	722.5**	109.05**	105.04**	29.571**	30.48**	45.54**

Values (Mean ± SE) with dissimilar letters in a column are significantly different at **p < 0.01.

**Figure 6:**
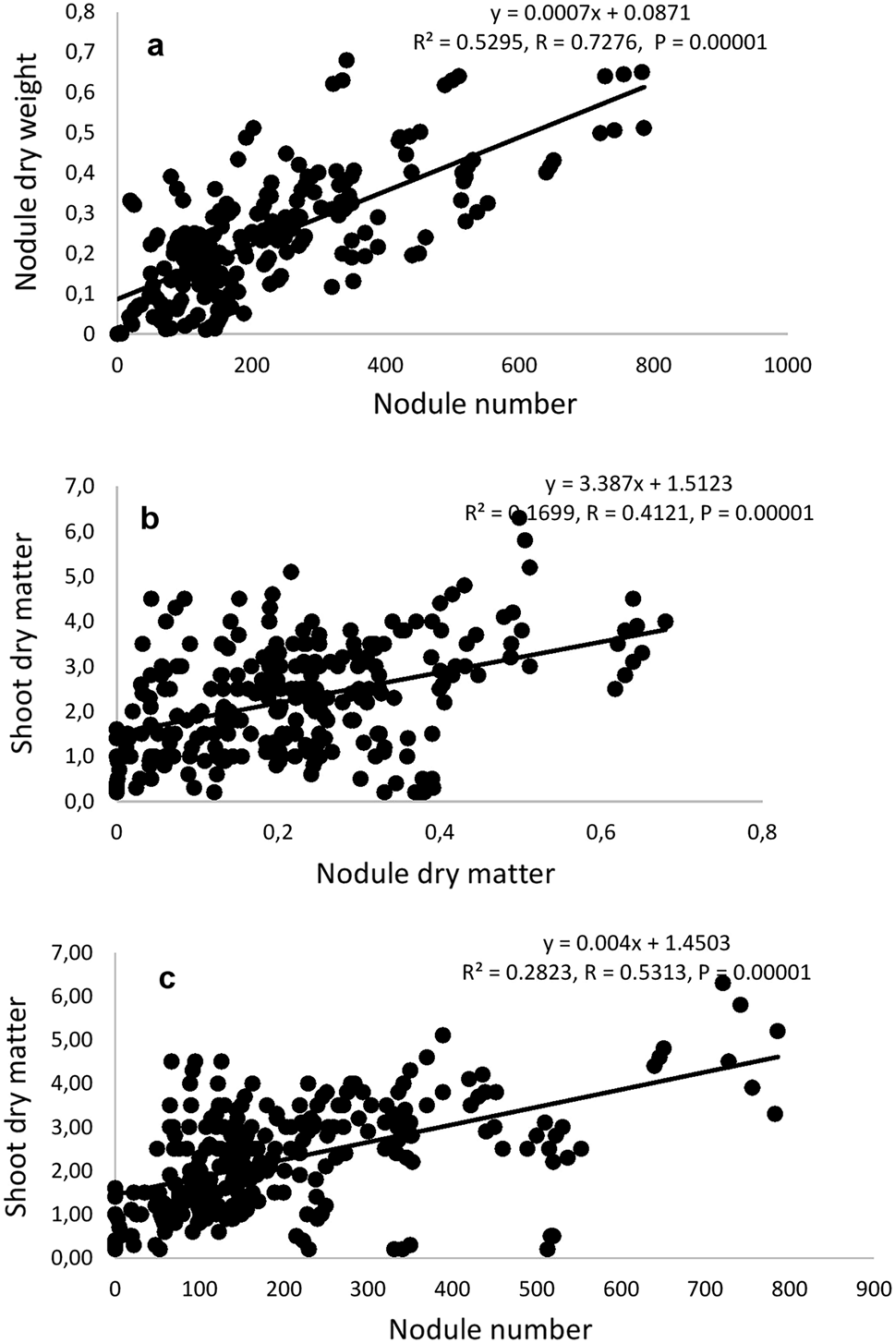
Correlation between (**a**) Nodule number and nodule dry weight (**b**) nodule dry weight and shoot dry matter (**c**) nodule number and shoot dry matter.

Nevertheless, there was a significant positive correlation between nodule number and nodule dry matter induced by the isolates (Fig. 6a). Generally, the rhizobia that induced higher nodule numbers, also elicited greater shoot dry matter in the common bean host plant, with a few exceptions. There was therefore a significant positive correlation when nodule number and nodule dry matter were each plotted against shoot DM (Fig. 6b and c). The values of shoot DM ranged from 0.23 g. plant^-1^ in plants inoculated with isolate TUTPvES 53(4) to 5.77 g. plant^-1^ in plants inoculated with isolate TUTPvES 20(1). Of all tested isolates, 65 induced significantly higher shoot biomass than the nitrate fed plants, whereas 24 of the isolates induced lower shoot biomass than the nitrate fed plants.

Relative symbiotic effectiveness (RE) ranged from 18% in the plants inoculated with isolate TUTPvES 53(4) to 433% in the plants inoculated with isolate TUTPvES 20(1). The data showed that over 85% of the isolates were highly effective (with RE between 103 and 433%) while five isolates were less effective (Table 2).

### Photosynthetic rates induced by rhizobial isolates

Inoculating common bean seedlings with the different rhizobial isolates induced varying levels of leaf photosynthetic rates (A), and other gas exchange parameters in the plant. Plants that were inoculated with isolates TUTPvES 24(1), TUTPvES 2(3), TUTPvES 5(1) and TUTPvES 11(3) induced significantly higher photosynthetic rates (20.71, 20.61, 20.60 and 20.47 µmol (CO_2_) m^-2^ s^-1^, respectively) than those inoculated with the remaining isolates (Table 2). With regards to stomatal conductance, plants that were inoculated by isolates TUTPvES 49(2), TUTPvES 6(1), TUTPvES 59(1) and TUTPvES 11(3) induced significantly higher values (0.45, 0.45, 0.44 and 0.42 µmol (CO_2_) m^-2^ s^-1^, respectively) than those inoculated with the remaining isolates. With few exceptions however, the isolates which induced lower stomatal conductance and transpiration rates (E) were generally found to induce lower photosynthetic rates in the host common bean, and vice versa (Table 2).

## Discussion

### Morpho-genetic diversity of rhizobial symbionts of common bean

While the diversity of rhizobia nodulating common bean has been studied worldwide^9,11^, little is known about the crop’s rhizobial microsymbionts in Swati (of Eswatini) soils. The differences in morphological features of bacteria can be used as a preliminary assessment of their diversity. In this study, the diversity of rhizobial symbionts of common bean from Eswatini was evidenced by differences in their growth rate and colony appearance. However, because distantly related rhizobia may share similar morpho-physiological characteristics, ERIC PCR fingerprinting is often used as a robust tool in distinguishing closely related species^12^.

The genetic diversity of common bean rhizobia in Swati soils was further assessed using ERIC-PCR fingerprinting. The dendrogram constructed from ERIC-PCR profiles placed a total of 88 common bean isolates in 11 major clusters when considering a 20% similarity level, which represented 80 ERIC PCR types if considered at a 70% similarity level. The diversity of bacteria nodulating various legumes, including common bean, have previously been analysed using ERIC-PCR profiles^13^. For example, the study by Zinga et al.^9^ observed high genetic diversity among rhizobial symbionts of common bean in South African soils. Although the number of authenticated rhizobia in this study varied between common bean genotypes, the isolates from genotype DAB 381 showed greater diversity, as they [e.g., TUTPvES 4(1), TUTPvES 4(2), TUTPvES 4(3) and TUTPvES 4(4)] were distributed in four different ERIC clusters (i.e., cluster II, IV, VII and X) (Fig. 1). Similarly, the isolates from genotype DAB 155 appeared in three of the clusters. Within the major clusters, there was a general tendency for isolates from the same field or genotype to group closely together. These observations are consistent with earlier reports that similarly reported the clustering of rhizobial symbionts of cowpea in Mozambique based on their geographic location of origin^14^. As the fields used to trap rhizobia in this study were in proximity at the Malkerns Research Station, the soil chemical properties did not show marked variation; except for the levels of available P which was slightly higher in the soil planted to *SARBEN* common bean genotypes (Supplementary Table S2). It may therefore be worthwhile to explore the symbionts of the test legume across contrasting agroecologies of Eswatini to provide more insights into the impact of environmental variables on the diversity of the crop’s symbionts.

### Phylogenetic positions of rhizobia nodulating common bean in Eswatini

Common bean has been widely reported to be promiscuous to a wide range of rhizobia and can be nodulated by different species including *R. etli, R. mayense, R pusense, R lusitanum* and *R. fabae*^9,15^. The fields in Eswatini used in this study had no history of inoculation; thus, the rhizobial isolates obtained were considered indigenous to the area, even though common bean seeds may carry viable rhizobial cells from different geographic environments^16^. The rhizobial isolates in this study aligned with different species belonging to the *Rhizobium* genus based on the 16S rRNA gene sequence analysis. As shown in Fig. 2, some of the indigenous isolates shared close relations with *R. freirei, R. paranaense, R. pusense* and *R. tropici*. However, because the 16S rRNA gene tends to show low resolution at the species/genus level, different housekeeping genes are often used to decipher closely related species^17,18^. In this study, the phylogeny of rhizobial isolates based on the 16S rRNA gene was mostly congruent with those of the housekeeping genes (*dnaK*, *glnII*, *gyrB* and *rpoB*), with the test isolates aligning closely with *Rhizobium* type strains in the different phylograms. As a result, the phylogram based on concatenated *glnII* + *dnaK* + *rpoB* was also congruent with those of individual housekeeping genes and showed close similarity with the type strains *R. paranaense* and *R. pusense* (Fig. 3). However, although isolate TUTPvES 14(1) consistently aligned with *R. tropici* in the 16S rRNA phylogeny and in the phylograms based on the individual housekeeping genes, the isolate stood away from the other type strains in the concatenated gene phylogeny due to the absence of *R*. *tropici* in that tree. These findings are similar to those of Zinga et al.^9^ who also found close relatives of *R. tropici* in the root nodules of common bean in South African soils.

Interestingly, although *R. phaseoli* and *R. etli* are known symbionts of common bean^19^, the indigenous isolates in this study showed low similarity with those type strains. *Rhizobium tropici* is a well-known microsymbiont for common bean and has been reported as the main symbiont of the crop in Latin America and in East, West and southern Africa^20^. However, as observed in this study, *R. paranaense* has previously been isolated from root nodules of common bean in sub-Saharan Africa^20^. The other test isolates which did not align closely with the known type strains will require a detailed description via whole genome sequencing.

As to be expected, the phylograms based on the two symbiotic genes (*nifH* and *nodC*) were incongruent with the 16S rRNA and the housekeeping genes phylogenies. For example, although the isolates grouped in different clusters in the housekeeping gene phylogenies, they formed a single cluster in the *nifH* and *nodC* phylogenies, an observation that could be attributed to the acquisition of those genes via horizontal gene transfer from a common ancestor since they are located on transmissible plasmids in some *Rhizobium* species^21,22^. A similar observation was made by Zinga et al.^22^ in a study on common bean symbionts from South Africa and Mozambique. Aserse et al.^23^ also reported incongruency between the phylogenies inferred from two symbiotic genes and that of the 16S rRNA gene in Ethiopia due to possible inter strain gene transfer and gene recombination.

Dlamini et al.^24^ had earlier reported the influence of soil pH and nutrient levels of the distribution of rhizobia associated with Bambara groundnut from different locations in Eswatini. Since the present study focused on the symbionts of common beans grown at one location, it may be worthwhile to explore the diversity of the crop’s symbionts across contrasting agroecologies of Eswatini in subsequent works.

### Symbiotic efficiency of common bean isolates from Eswatini

Aside from variability in morpho-genetic characteristics, the diversity of common bean isolates in this study were also shown by the variable nodulation, plant growth and photosynthetic parameters they elicited in the host plants. Whereas some isolates induced both greater nodule number and nodule weight in the host, others elicited greater nodule weight despite inducing fewer nodules in the host; these observations were probably due to differences in nodule size as well as the N_2_-fixing efficiency of the rhizobial symbionts^25^. For example, despite forming few nodules, isolate TUTPvES 45(1) induced higher nodule dry matter, which led to higher photosynthetic rates and shoot dry matter when compared to the 5 mM nitrate fed plants. In some instances, nodule symbionts elicited lower plant growth and photosynthesis in the host despite inducing high nodulation, an indication that some of the nodule symbionts were less effective in fixing nitrogen (N_2_). Some studies have observed that legumes can sometimes form ineffective nodules that harbour low N_2_-fixing rhizobia^26^. Nevertheless, nodule number and nodule weight were both positively correlated with shoot biomass, indicating that the nodule symbionts contributed to plant growth promotion.

As the uninoculated control plants expectedly showed the least plant growth, the effectiveness of isolates was assessed by comparing the biomass of inoculated plants with that of nitrate fed plants^27^. Of the 88 isolates, 74% elicited greater shoot biomass in the host common bean when compared to the 5 mM nitrate-fed plants, with relative effectiveness values ranging from 103 to 433%. Thus, the soils in Eswatini are home to highly effective rhizobia that can potentially be used to formulate commercial inoculants for increased common bean cultivation upon extensive testing in the field to assess their adaptation to prevailing abiotic conditions.

## Conclusion

Based on their ERIC PCR banding patterns, this study revealed a high genetic diversity among common bean symbionts in Eswatini, an observation consistent with several reports on the diversity of rhizobia in African soils. Moreover, multilocus sequence analysis based on the sequences of 16S rRNA, *rpoB, dnaK, gyrB,* and *glnII* and symbiotic (*nifH* and *nodC*) genes aligned the test isolates with the type strains of *Rhizobium muluonense*, *R. paranaense*, *R. pusense, R. phaseoli* and *R. etli.* Some of the indigenous isolates showed a high divergence from the known reference type strains, and may require further description via whole genome sequencing. Aside from their diversity, a glasshouse experiment showed that most of the isolates were efficient in fixing nitrogen, and elicited greater stomatal conductance and photosynthetic rates in the common bean host. Relative symbiotic effectiveness (RE) of the isolates varied from 18 to 433%, with 75 out of the 80 tested isolates producing greater shoot biomass than the nitrate fed control plants.

## Materials and methods

### Nodule collection and bacterial isolation

Root nodules were collected from different common bean genotypes grown at the Malkerns Research Station in Eswatini during the 2017/2018 cropping season. The soil chemical properties as well as the climatic data of the Malkerns site during the 2017/2018 cropping season are presented in Supplementary Tables S1 and S2. For this, the plants were carefully dug out at the early podding stage and the nodulated roots separated from the shoots. The roots were then transported to the laboratory in prelabelled zip-lock plastics in a cooler box with ice. The roots were rinsed in running tap water to remove debris, and the nodules attached to small root segments removed and preserved on silica gel in plastic vials prior to bacterial isolation. Bacteria were isolated from the nodules according to the procedure described by Somasegaran and Hoben^28^. For this, surface sterilized nodules were crushed in a loop of sterile water in a petri dish, and the nodule macerate streaked on yeast mannitol agar (YMA) plates and incubated at 28 °C. The plates were monitored daily for colony appearance. The number of days taken for colonies to appear as well as other morphological characteristics (colony size/shape, colour, and texture) were recorded^29,30^.

### Nodulation bioassay in the glasshouse

A total of 162 bacterial isolates were obtained from the root nodules of the different common bean genotypes. To fulfil Koch’s postulates, single-colony cultures from these bacterial isolates were assessed for their ability to elicit root nodules on their homologous Kranskop common bean genotype in a naturally lit glasshouse under aseptic conditions. For this, the common bean seeds were surface sterilized as described by^29^ and planted in sterile (autoclaved) sand which were contained in sterile plastic pots. After germination, the seedlings were inoculated with 1 mL broth culture of the different bacterial isolates grown to the exponential phase, with three replicate pots per isolate. The plants were watered with autoclaved nitrogen-free nutrient solution and sterile distilled water in alternation. Uninoculated plants and plants treated with 5 mM KNO_3_ were used as negative and positive controls, respectively. At 60 days after planting, the plants were harvested and assessed for nodulation; isolates which elicited nodules on three replicate plants were considered as rhizobia. The use of plant materials in different aspects of this study complied with relevant institutional, national and/or institutional guidelines.

### Assessment of plant growth and photosynthetic rates induced by isolates

The authenticated rhizobia were further assessed for their symbiotic efficiency using plant nodulation, growth, and photosynthetic rates as reference parameters. For this, photosynthetic rates (A), stomatal conductance (gs) and transpiration rates (E) were measured on the youngest fully expanded trifoliate leaves of common bean plants inoculated with the different isolates using a portable infrared red gas analyser, version 6.2 (LI 6400XT, Lincoln, Nebraska, USA). The chamber conditions were set as follows: photosynthetic flux density of 1000 μmolm^-2^ s^-1^, reference CO_2_ concentration of 400 μmolmol^-1^ and flow rate of 500 μmols^-1^. The gas exchange measurements were performed between 9.00 am and 12.00 pm at 60 days after planting^31^. Thereafter, the plants were harvested, separated into shoots and nodulated roots, and the nodules collected from the roots. The shoots and nodules were separately oven-dried in brown paper envelopes, and weighed to determine shoot and nodule dry matter, respectively. The relative effectiveness (RE) of isolates was calculated by expressing the shoot biomass of plants inoculated with the rhizobial isolates as a percentage of the shoot biomass of plants treated with the 5 mM KNO_3_^32^.

$$\mathrm{RE }=\frac{\mathrm{shoot\,dry\,matter\,of\,innoculated\,plant}}{\mathrm{shoot\,dry\,matter\,of\,N}-\mathrm{fertilized\,plant }}\times 100$$

The isolates were categorized as highly effective (> 80% RE), moderately effective (50 to 80% RE), lowly effective (35 to 49% RE) and ineffective (< 35% RE).

### Molecular characterization of isolates

#### Genomic DNA extraction and ERIC PCR fingerprinting

Bacterial genomic DNA extraction was carried out using the GenElute bacterial DNA isolation kit by following the manufacturer’s instructions (Sigma Aldrich, USA). The quality of DNA was assessed on a 1% agarose gel stained with ethidium bromide. The genomic DNA of rhizobial isolates were subjected to ERIC-PCR fingerprinting. The final PCR reaction volume was 25 μL and contained 1 μL of genomic DNA,12.5 μL 2× myTaq PCR master mix (Bioline USA), 1 μL each of the forward and reverse ERIC primers and 9.5 mL double distilled water. The DNA amplification was carried out in a Thermal cycler (T100 Bio-Rad, USA) using standard temperature profiles^24,33^, and the PCR products were subjected to gel electrophoresis on a 1% agarose gel at 85 V for 5 h. A cluster analysis to determine the similarities among isolates using the Jaccard’s similarity coefficient was performed with the Bionumerics software (version 8.1).

#### PCR amplification of 16S rRNA, housekeeping (*rpoB*, *dnaK*, *gyrB*, and *glnII*) and symbiotic (*nifH* and *nodC*) genes

The amplification of the 16S rRNA, housekeeping genes (*rpoB, dnaK, gyrB,* and *glnII*), and symbiotic (*nifH* and *nodC*) genes were carried out in a 25 μL PCR reaction mixture which contained 1 μL bacterial DNA, 3 μL of 5× myTaq buffer, 1 μL each of the forward and reverse primers of the gene of interest, 0.1 μL Taq polymerase (Bioline, USA), and 18.9 μL sterile ultrapure water using standard temperature profiles (Supplementary Table S3) in a T100 Bio-Rad Thermal Cycler, USA. The amplified gene products were confirmed by gel electrophoresis in a 1% agarose gel and the image captured using the Geldoc^Tm^ XR + system (Bio-Rad, USA). The primers used and their temperature profiles are shown in Supplementary Table S3.

#### Sequencing and Phylogenetic analysis of amplified genes

The PCR-amplified gene products were purified using a PCR cleanup kit (NEB, USA) following the manufacturer’s instructions. Purified DNA was sequenced at Macrogen laboratories (The Netherlands). The software BioEdit 7.0.9.0 was used to confirm the quality of sequences^34^. The sequences of each gene were subjected to BLASTn in the National Centre Biotechnology Information (NCBI) database to identify closely related rhizobial species. The alignment of the reference strain sequences with the test rhizobial isolates were done with CLUSTAL W and phylogenetic trees were inferred using MEGA 7 software^35^. The Kimura 2-paramete model with uniform rates among the sites was used to calculate evolutionary distances and evolutionary history was inferred using the maximum likelihood method. The robustness of tree branching was estimated using 1000 bootstrap replicates of the sequence^36^.

### Statistical analysis

All quantitative data collected from the glasshouse experiment were subjected to a one-way ANOVA using the STATISTICA program (Version 10). The quantitative datasets such as nodule number, nodule dry matter, shoot dry matter, photosynthetic rates (A), stomatal conductance (gs), leaf transpiration (E) and relative symbiotic effectiveness (RE) were tested for normality by calculating skewness and kurtosis values using the Data Analysis component of Excel. The skewness and kurtosis values ranged from − 0.10 to + 1.51 and −1.67 to + 2.30, respectively, and are consistent with values of a normal distribution^37^. Where there were significant treatment differences, the Duncan’s multiple range test was used to separate the means at *p* ≤ 0.05. Correlation analyses were done to assess the relationship between measured parameters.

### Supplementary Information

Supplementary Information.

## Data Availability

The nucleotide sequences of all the tested genes were submitted to the NCBI GenBank database to obtain the accession numbers: OP537152–OP537165 (16S rRNA), OP654835–OP654843 (*gyrB*), OP654807–OP654820 (*dnaK*), OP654821–OP654834 (*glnII*), OP654844–OP654855 (*rpoB*), OP654856–OP654868 (*nifH*) and OP654869–OP654872 (*nodC*).
