# Supplementary material for: Phylogeny and symbiotic effectiveness of indigenous rhizobial microsymbionts of common bean (Phaseolus vulgaris L.) in Malkerns, Eswatini
Source: Sci Rep. 2023 Oct 9;13:17029. doi: 10.1038/s41598-023-43634-5 (PMC10562383; doi:10.1038/s41598-023-43634-5)
Supplement: Supplementary file 1 — Supplementary Information. [file 41598_2023_43634_MOESM1_ESM.pdf]

Phylogeny and symbiotic effectiveness of indigenous rhizobial microsymbionts of common bean (*Phaseolus vulgaris* L.) in Malkerns, Eswatini

Rotondwa P. Gununununu[1], Mustapha Mohammed[3], Sanjay K Jaiswal[2], Felix D Dakora[2]

[1]Department of Crop Sciences and [2]Department of Chemistry, Tshwane University of Technology, Private Bag X680, Pretoria, 001, South Africa

[3]Department of Crop Science, University for Development Studies, P.O. Box TL1882, Tamale, Ghana

Orcid ID

Rotondwa P. Gununununu**:** https://orcid.org/0000-0002-1083-969X

Mustapha Mohammed: https://orcid.org/0000-0002-1415-1294

*Corresponding author:

Felix D. Dakora

Chemistry Department, Tshwane University of Technology, Private Bag X680, Pretoria 0001, South Africa.

Tel: +27 12 382 6120

Fax: +27 12 382 6286

Email: DakoraFD@tut.ac.za

Supplementary Table S1: Monthly climate data during the 2017/2018 cropping season

	Temperature (°C)		Rainfall (mm)	Relative humidity (%)	
**2017/2018**	**Min.**	**Max.**		**Min.**	**Max.**
December	16.9	26.8	228.6	66.6	95.8
January	16.9	28.9	100	55.6	95.5
February	18.3	28.3	175	64	96.8
March	16.8	28.5	200	62.4	97.8
April	15.7	26.7	76.6	62.6	97.8
May	16.92	25.3	36.2	51.9	96.7
**Mean**	**16.9**	**27.4**	**136.1**	**60.5**	**96.7**

Supplementary Table S2: Chemical properties of soils sampled from fields E, F and S at the Malkerns Research Station during the 2017/2018 cropping season.

Field	pH	Total N	Available P	Na	K	Ca	Mg	CEC
		**%**	**mg. kg^{-1}**	**mg. kg^{-1}**	**mg. kg^{-1}**	**mg. kg^{-1}**	**cmol (+).kg^{-1}**	**cmol (+).kg^{-1}**
**E**	4.9	0.062	11	0.044	0.338	1.966	1.209	5.443
**F**	4.5	0.062	22	0.033	0.286	1.392	0.831	4.049
**S**	4.8	0.052	9	0.036	0.343	1.976	1.193	5.685

* E = Field planted to *SARBYT* genotypes, F = field planted to *SARBEN* genotypes, S = field planted to *Sugar Nursery* genotypes

Supplementary Table S3: Primers and temperature profiles used for PCR amplification of the various genes.

Primer	Sequences 5'-3'	Temperature profile	Reference
Eric F	ATGTAAGCTCCTGGGGATTCAC	5 min 95 °C, 30 × (30 s 94°C, 1 min 52 °C, 8 min 65°C), 16 min 65 °C	Versalovic *et al*. 1991
Eric R	AAGTAAGTGACTGGGGTGAGCG		
16SrRNA F	AGAGTTTGATCCTGGCTCAG	2 min 95 °C, 30 X (15 s 94 °C, 45 s 93°C, 45 s 55°C, 2 min 72°C) 5 min 72°C	Weisburg *et al*. 1991
16SrRNA R	CTTAAGGAGGTGATCCAGCC		
gyrB F	TTCGACCAGAAYTCCTAYAAGG	10 min at 95°C, 35 X (30s at 94°C, 30s at 58°C, 1 min at 72°C), 10 min at 72°C	Marek-Kozaczuk *et al*. 2013
gyrB R	AGCTTGTCCTTSGTCTGCG		
glnII F	AAGCTCGAGTACATCTGGCTCGACGG	2 min at 95°C, 35 X (45s at 95°C, 30s at 65°C, 90s at 72°C), 10 min at 72°C	Stepkowski *et al*. 2011
glnII R	SGAGCCGTTCCAGTCGGTGGTGTCG		
rpoB F	ACATCGAGTTCGACGCCAAGG	5 min at 95°C; 20 x 45s at 95°C, 30s at 60°C (-0.5°C per cycle) and 1 min 30s	Nzoué *et al*. 2009
rpoB R	CATTGACGTGGTCGATGTCG	at 72°C; 25 x 30s at 94°C, 30s at 55°C, 1 min 30s at 72°C; 10 min at 72°C	
dnaK F	GTACATGGCCTCGCCGAGCTTCA	1 min 94°C, 35 x (1 min 94°C, 1 min 62°C, 40 s 72°C)	Stępkowski *et al*. 2003
dnaK R	AAGGAGCAGCAGATCCGCATCCA		
*nod*C F	GTCGATTGCMRGTCAAGACTACG	30s at 94°C, 40× [30s at 94°C, 1 min at 55.4°C, 30s at 72°C], 5 min 72°C	Laguerre *et al*. 2001
*nod*C R	GCCAGGTCTIGTTGCGATTGCTC		
nifH F	TACGGNAARGGSGGNATCGGCAA	5 min at 94°C, 20 X [30s at 94°C, 30s at 65°C (-0.5°C per cycle), 90s at 72°C],	Nzoué *et al*. 2009
nifH R	AGCATGTCYTCSAGYTCNTCCA	25 x (30s at 94°C, 30s at 55°C, 90s at 72°C), 10 min at 72°C	

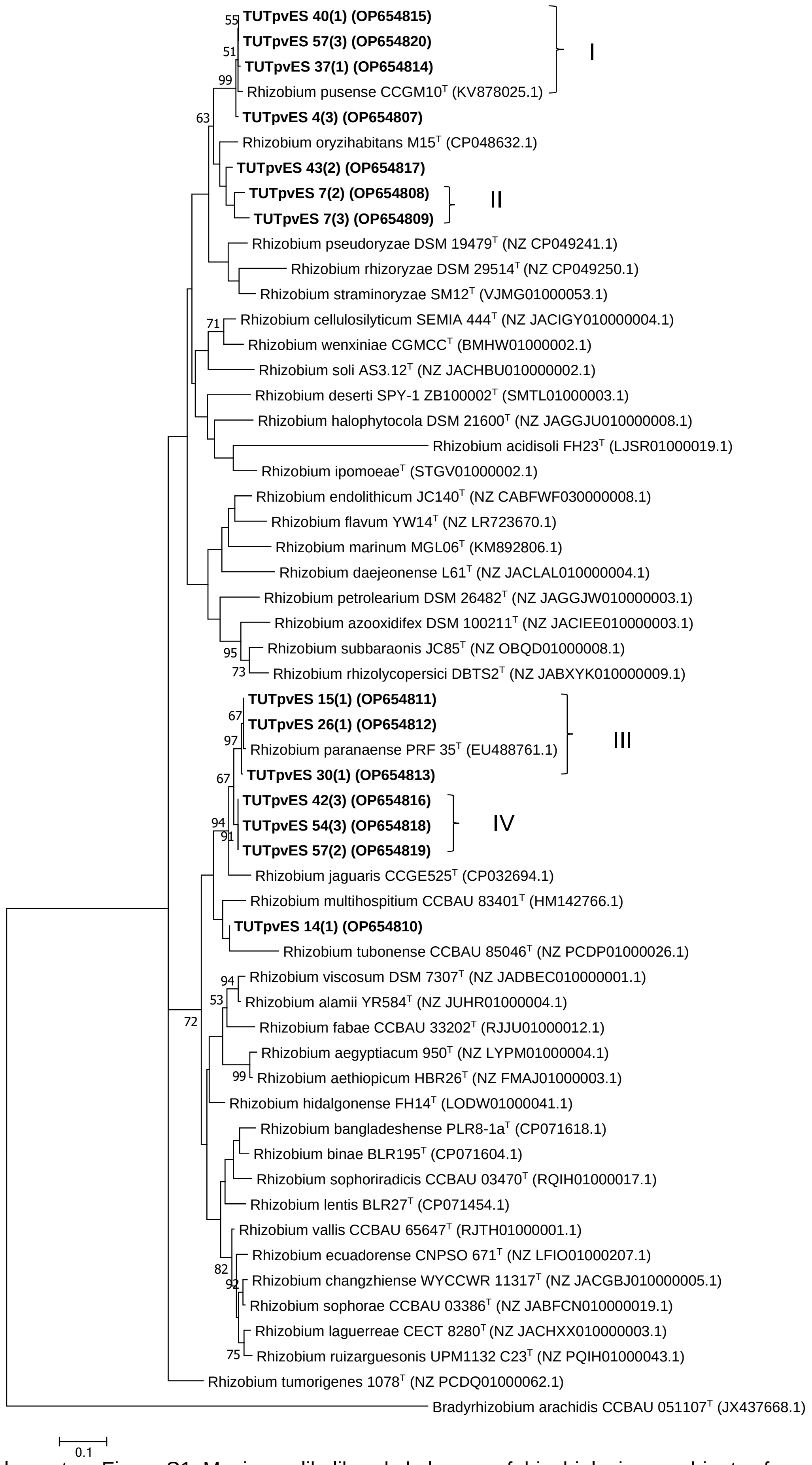

Supplementary Figure S1: Maximum-likelihood phylogeny of rhizobial microsymbionts of common bean from Eswatini inferred from *dnaK* gene sequences. Phylogenetic trees were inferred using MEGA 7 software. The Kimura 2-paramete model with uniform rates among the sites was used to calculate evolutionary distances.

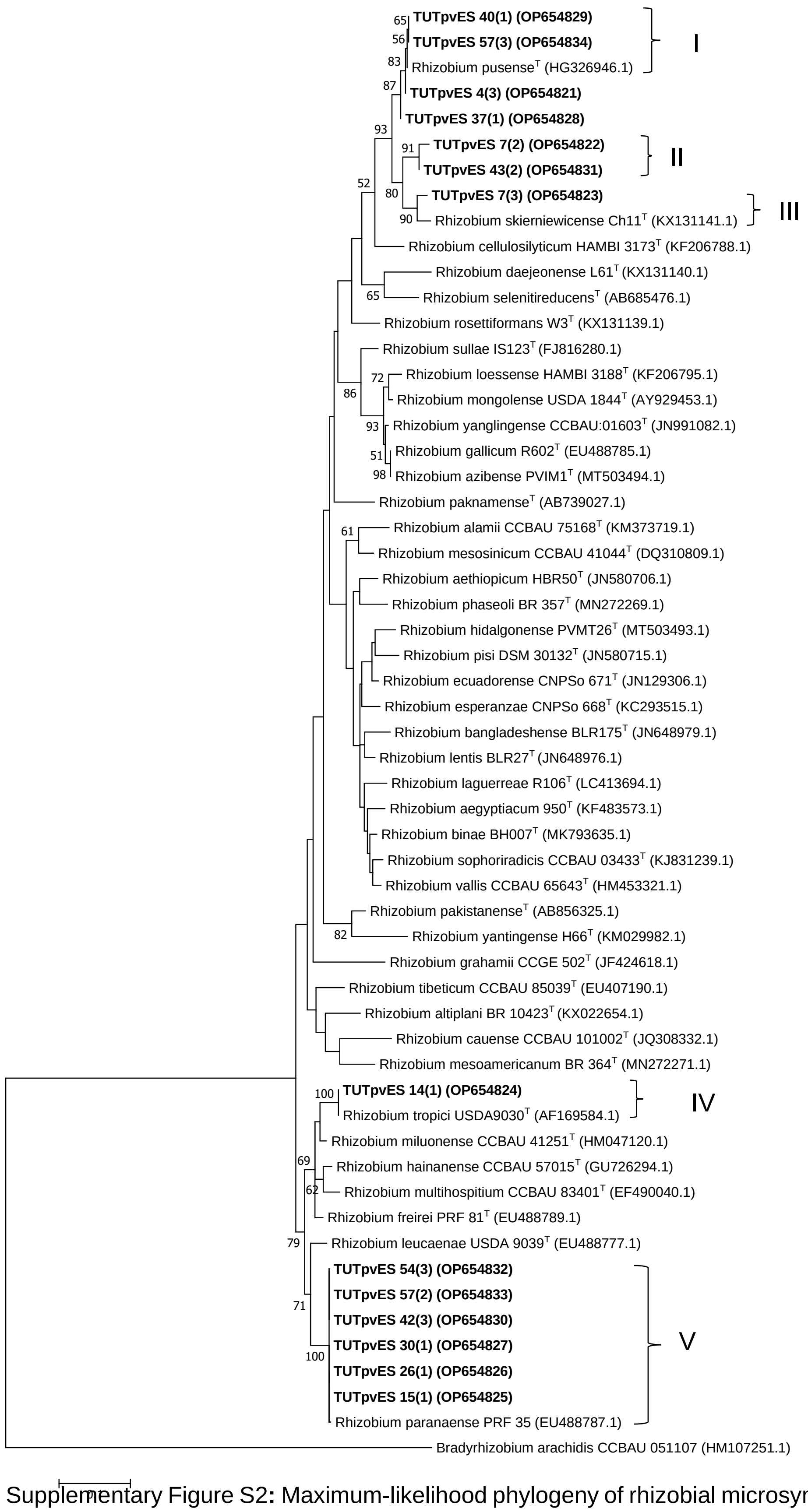

Supplementary Figure S2**:** Maximum-likelihood phylogeny of rhizobial microsymbionts of common bean from Eswatini inferred from *glnII* gene sequences. Phylogenetic trees were inferred using MEGA 7 software. The Kimura 2-paramete model with uniform rates among the sites was used to calculate evolutionary distances.

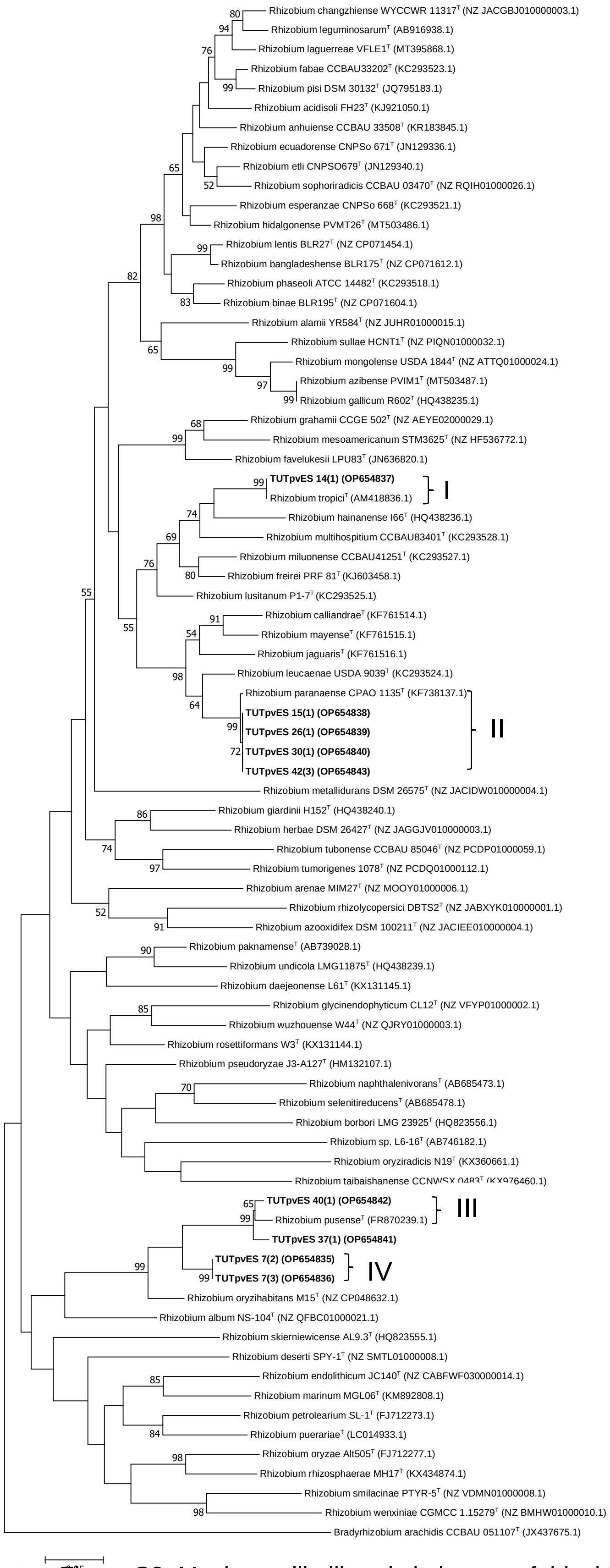

Supplementary Figure S3**:** Maximum-likelihood phylogeny of rhizobial microsymbionts of common bean from Eswatini inferred from *gyrB* gene sequences. Phylogenetic trees were inferred using MEGA 7 software. The Kimura 2-paramete model with uniform rates among the sites was used to calculate evolutionary distances.

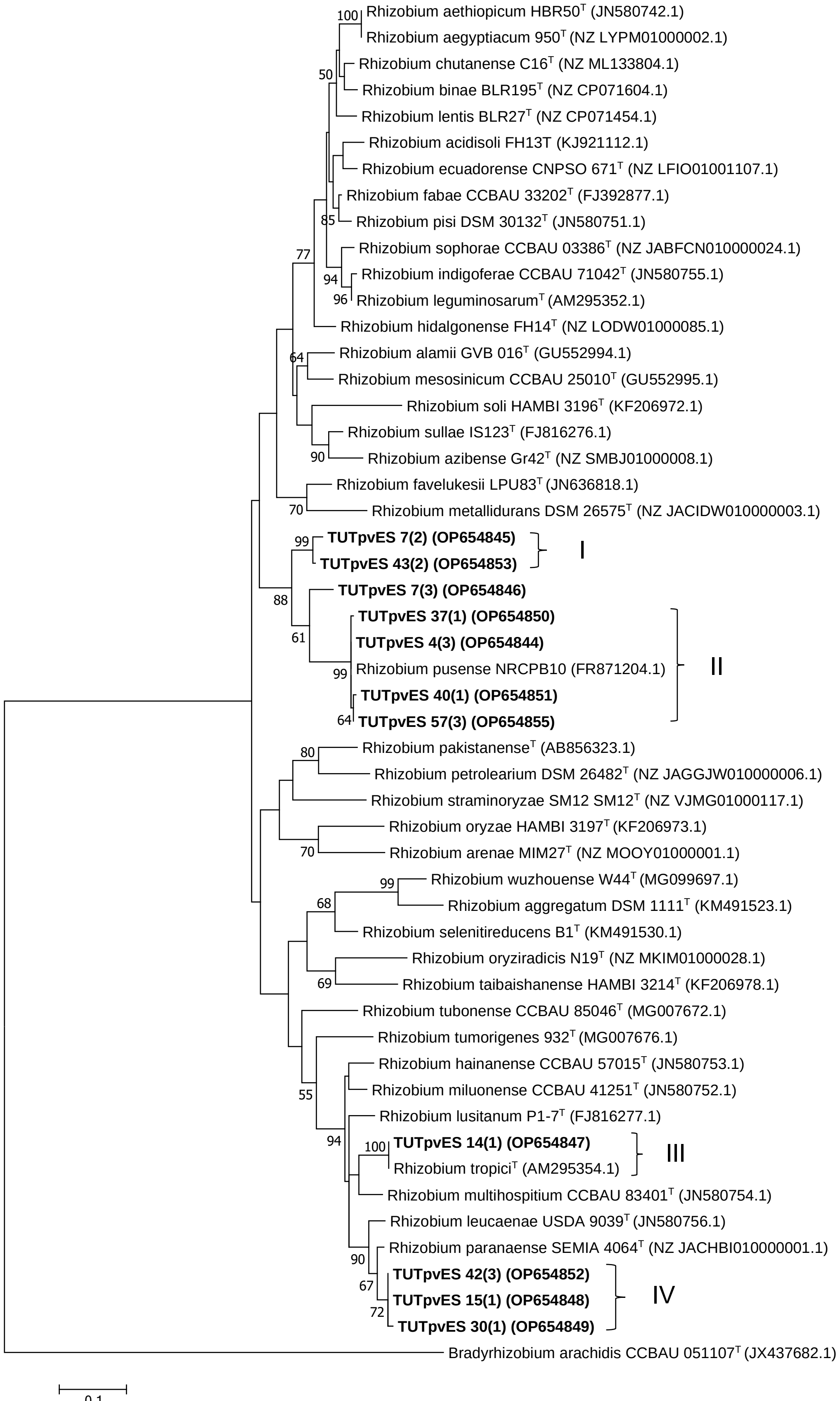

Supplementary Figure S4**:** Maximum-likelihood phylogeny of rhizobial microsymbionts of common bean from Eswatini inferred from *rpoB* gene sequences. Phylogenetic trees were inferred using MEGA 7 software. The Kimura 2-paramete model with uniform rates among the sites was used to calculate evolutionary distances.
